# Possible explanations for why some countries were harder hit by the pandemic influenza virus in 2009 – a global mortality impact modeling study

**DOI:** 10.1186/s12879-017-2730-0

**Published:** 2017-09-25

**Authors:** Kathleen F. Morales, John Paget, Peter Spreeuwenberg

**Affiliations:** 1 Sage Analytica, Portland, Maine USA; 2 Netherlands Institute for Health Services Research (NIVEL), Utrecht, The Netherlands

**Keywords:** Influenza, Pandemic, Mortality, Modelling, Risk factors, Global

## Abstract

**Background:**

A global pandemic mortality study found prominent regional mortality variations in 2009 for Influenza A(H1N1)pdm09. Our study attempts to identify factors that explain why the pandemic mortality burden was high in some countries and low in others.

**Methods:**

As a starting point, we identified possible risk factors worth investigating for Influenza A(H1N1)pdm09 mortality through a targeted literature search. We then used a modeling procedure (data simulations and regression models) to identify factors that could explain differences in respiratory mortality due to Influenza A(H1N1)pdm09. We ran sixteen models to produce robust results and draw conclusions. In order to assess the role of each factor in explaining differences in excess pandemic mortality, we calculated the reduction in between country variance, which can be viewed as an effect-size for each factor.

**Results:**

The literature search identified 124 publications and 48 possible risk factors, of which we were able to identify 27 factors with appropriate global datasets. The modelling procedure indicated that age structure (explaining 40% of the mean between country variance), latitude (8%), influenza A and B viruses circulating during the pandemic (3–8%), influenza A and B viruses circulating during the preceding influenza season (2–6%), air pollution (pm10; 4%) and the prevalence of other infections (HIV and TB) (4–6%) were factors that explained differences in mortality around the world. Healthcare expenditure, levels of obesity, the distribution of antivirals, and air travel did not explain global pandemic mortality differences.

**Conclusions:**

Our study found that countries with a large proportion of young persons had higher pandemic mortality rates in 2009. The co-circulation of influenza viruses during the pandemic and the circulation of influenza viruses during the preceding season were also associated with pandemic mortality rates. We found that real time assessments of 2009 pandemic mortality risk factors (e.g. obesity) probably led to a number of false positive findings.

**Electronic supplementary material:**

The online version of this article (10.1186/s12879-017-2730-0) contains supplementary material, which is available to authorized users.

## Background

Since the first confirmed death of Influenza A(H1N1)pdm09 in Mexico in April 2009 [1], researchers and public health officials continue to search for clarity on the global morbidity and mortality impact of Influenza A(H1N1)pdm09 and its associated risk factors, in order to improve future pandemic response. At the start of an influenza pandemic, known burden statistics, mostly relating to seasonal influenza, are used to anticipate burden, target pandemic response, and identify high-risk groups. This can lead to confusion when real pandemic data slowly comes in and contradicts this paradigm, leading to stymied response activities as the overall risk and factors to mitigate that risk are poorly understood. We use a standardized estimation methodology to contribute to the evidence base of the global impact of Influenza A(H1N1)pdm09 and its risk factors. Our results and approach can be used to provide guidance for future pandemic response.

Numerous studies have aimed to capture the global mortality impact of Influenza A (H1N1)pdm09 and identify factors to explain mortality variations seen across populations. Studies have focused on familiar risk factors for seasonal influenza, such as comorbidities [2–14], age [15–19], pregnancy [20–22], healthcare-related factors [23, 24], climate [10, 25–28], and treatment approaches [3, 29–34], while others have explored more obscure risk factors including pollution exposure [28], pandemic preparedness activities [30, 35], international flight travel [36], viral shedding [8, 26, 37], and pandemic timing [30, 36, 38, 39]. These studies have three limitations: 1.) They are typically executed in local hospital or community settings, limiting the opportunity for comparisons across greater communities or countries; 2.) The number of factors studied is limited – typically only focusing on a few at once- comorbidities, physiological factors, or climate for example - not looking at all together; 3.) The definition and method of calculation for influenza mortality varies, further limiting comparison across studies.

Very few studies have looked at risk factors in multiple countries [40] and no studies, to our knowledge, have addressed the above weaknesses. Our study does this by: a.) Investigating the global Influenza A (H1N1)pdm09 mortality impact within and across all world countries and the associated risk factors; b.) Including an exhaustive list of possible risk factors believed to be associated with seasonal and/or pandemic influenza; c.) Using standardized data collection techniques and source data for both mortality and risk factors to ensure comparability across countries. By filling these gaps and strengthening the data on mortality and risk factors, we hope to further the evidence base of pandemic H1N1 risk factors.

The GLaMOR [41] study utilized a standardized approach to estimate country-specific mortality rates in 200 countries in 2009. Through this approach, the study found a mortality burden 20× higher than the WHO estimate based on laboratory confirmed deaths from May 2010 [42]. In addition, the study found prominent regional mortality variations, proving the highest burden in the Americas and the lowest burden in Europe, an alternative to a study which found the highest burden in Africa and South-East Asia [43].

Inspired by the GLaMOR study’s findings and the standardized method utilizing global data, we combine the approach, including additional data on risk factors to determine the contribution of each risk factor on observed regional variations. In addition, we are able to confirm or dispute the impact of risk factors long believed to influence influenza mortality, such as pregnancy and obesity. This is the first study to investigate the role of multiple risk factors for H1N1 mortality at the global level using country-specific data in multiple countries.

## Methods

### Risk factor literature search

As a starting point, we identified possible risk factors worth investigating for Influenza A(H1N1)pdm09 mortality through a targeted literature search in Pubmed, focusing on 2000–2013. Chosen search terms included, “pH1N1 influenza mortality”, “pandemic influenza deaths”, “pandemic H1N1 risk factors”, “pandemic influenza severity”, “pandemic influenza outcomes”, “influenza risk factors”, and numerous variations on those search terms. Papers solely evaluating risk factors for Influenza A(H1N1)pdm09 as well as those comparing risk factors for seasonal influenza or previous influenza pandemics with Influenza A(H1N1)pdm09 were included. In addition, papers speculating on certain risk factors were also included as long as there was sufficient and plausible justification for the assumption.

Identified risk factors were then categorized into areas such as “physiological”, “environmental”, and “pandemic preparedness” as examples. Using these risk factors to guide the search, we then set out to identify global, public data sources, which contained comprehensive country-specific data for the whole world, such as WHO, the World Bank, and the UN.

### Risk factor data sources

Of the possible risk factors captured during the literature search, we identified appropriate data for approximately half of the factors. Criteria used to determine if data for each factor were sufficient for our approach were the following: a.) Identify enough data from one global source for at least 50 countries, b.) Data identified had to include data for all (most) 20 countries included in the first step in our model (explained below), c.) Data identified had to include countries from all continents and WHO regions, to ensure it was regionally diverse and not biased to one region d.) Data identified had to give values, which varied, determined through mean, variance, and standard deviation calculations.

Data sources for the factors in the analysis included the WHO Global Health Observatory Data Repository [44], the World Bank open data database [45], the WHO FluNet database [46], the IMS Health Antiviral purchasing database 2007–2009 [31], and the Global Asthma Report 2011 [47].

### Modelling

The global estimates of pandemic mortality in 2009 were based on 20 observed country excess mortality rates (these were modelled estimates based on weekly observed data) and 170 country estimates based on a simulation approach [41].

We used the GLaMOR modelling approach to compare the relation between a risk factor and the excess pandemic mortality under two conditions: the factor is present and the factor is absent. Because 170 country excess rates are calculated based on a simulation procedure, it is easy to calculate the excess rates under these two conditions (present or absent in the data creation process). The approach is similar to comparing a control group with an experimental group.

The full procedure can be summarized as follows:

- Step 1: Identify the potential risk factors that will be tested in the model – literature review and the direction of the effect (see above)
- Step 2: Generate data for every country with missing pandemic mortality estimates using the GLaMOR simulation approach [41] twice:
  1. Without the potential risk factor of interest e.g. total health expenditure
  2. With the potential risk factor of interest e.g. total health expenditure
- Step 3: Assess whether there is an association between the risk factor and the GLaMOR generated pandemic mortality data
  - For all countries, regress (=3) the risk factor with the GLaMOR generated data from (1)
  - For all countries, regress (=4) the risk factor with the GLaMOR generated data from (2)
- Step 4: Examine the relation of the risk factor with the pandemic mortality data
  1. Check how the regression coefficients vary between (3) and (4)? For example, if (3) is not significant and (4) is significant, the risk factor may be significant (see the Decision Rule Set (see: Additional file 1 - Technical Appendix) for all possibilities)
  2. Check the direction of the association (the regression coefficient 4)) based on the published literature. If the direction is wrong, then the association of the risk factor is considered to be not a determinant (association only)
  3. Check the association is not driven by age (as age is associated with many of the factors that are being assessed). This is done by re-running the regressions with the two age confounders (% under 15 and % over 60) to control for these artifacts. If the association remains it is considered a determinant and if it disappears (see Additional file 1 - Technical Appendix for procedure) the risk factor has no association
- Step 5: Repeat the steps 2, 3, 4 16 times using different data generation procedures (see below, ‘Robustness assurance’)
- Step 6: Credibility of the association for each risk factor is established by looking at the number of times a relation (no effect, association or determinant) is found. The relative effect is established by looking at the average reduction in the between country variance. (see below, ‘*Analysis of pandemic mortality, between country variance’*)

This procedure fits very well within Rubin’s Causal Inference Model [48], a model which has been used in a number of public health studies [49, 50]. Central to this approach is an outcome comparison under two conditions with and without the risk factor, and we have created a Decision Rule Set to classify each factor as: no effect, association effect or determinant effect (see Additional file 1).

### Robustness assurance

**Fig. 1:**
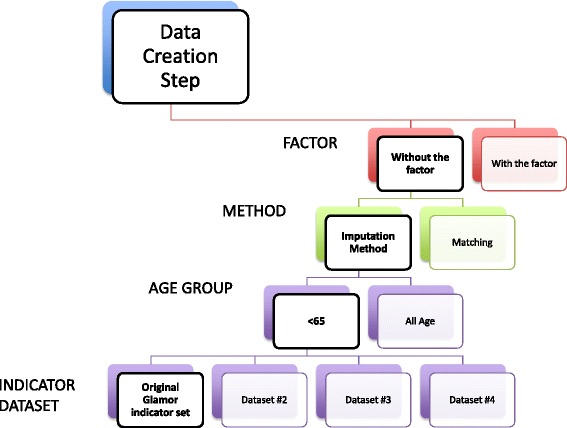
Outline of the 16 different GLaMOR modeling approaches with a selection of the method, the age and the dataset

For the GLaMOR study [41], the mortality estimation step using the imputation method was run twice, once for the age group <65 and once for all ages, so there were two results per country. For the current analysis, in order to ensure robust results and conclusions, we ran 16 different variations of the GLaMOR modeling approach: two different algorithms for the data creation step - the imputation and matching methods, two age groups (<65 and all ages) and four different predictor variables sets (see Fig. 1 and Additional file 1). We took this approach to minimize the possibility of identifying false or spurious effects.

### Analysis of pandemic mortality, between country variance

In order to assess the relative strength of each factor in explaining differences (an effect size) in excess pandemic mortality, we calculated the reduction in the between country mortality variance for each factor. This proportion is a measure of how strong a factor is in capturing the difference in country variance. It is calculated by comparing two regressions, based on the datasets in which the factor was present during the data creation stage. First the between country variance for the model without the factor in the regression model and next the between country variance with the factor in the model. The between country variance for the model with the factor present is then subtracted from the between country variance for the model without this factor. This is divided by the between country variance for the model without this factor and multiplied by 100 resulting in a percentage. If the percentage is 100%, then all the differences in excess mortality rates between countries could be explained by this factor, if it is 0% this factor cannot explain any difference between countries. This was done 16 times and the average is reported. So the effect size for a factor is the average reduction in between country variance.

## Results

### Risk factor literature search

The literature search captured 124 publications and identified 48 possible risk factors (Table 1) worth exploring. The 48 possible risk factors fall into 10 factor categories – environmental, comorbidities in adults and children, treatment, viral, healthcare, demographic, data or modeling, pandemic preparedness activities, and other.

**Table 1:** The 48 possible risk factors worth investigating for Influenza A(H1N1)pdm09 mortality through a targeted literature search in Pubmed (with a focus on 2000–2013)^a^

Factor type	Factor	Factor variable	Association higher^a^	Association lower^a^	References
Environmental	Pollution	Particulate matter	✓		[28]
		Ozone	✓		
	Climate	Low temperature/low humidity	✓		[10, 25, 26]
Physiological comorbidities in adults	Immunosuppression other than HIV	renal disease, chronic disease, cancer, etc.	✓		[5]
	Obesity	BMI > 30	✓		[3, 7]
	Morbid Obesity	BMI > 40	✓		[44]
	Pulmonary Disease	COPD, other	✓		[5]
	Immunosupression	HIV	✓		[57]
		TB	✓		
		HIV on ARTs		✓	[57]
Physiological comorbidities in children	Neurological Disorders	Prevalence in children	✓		[11]
	Cerebral Palsy and developmental delay		✓		[13]
	Congenital Heart Disease		✓		[13]
	Asthma and immunosupression		✓		[30, 40]
Treatment	Antiviral Use	Antiviral Drug Distribution		✓	[31]
		Antiviral within 48 h.		✓	[23, 28, 30]
		Late onset antiviral	✓		[3, 31, 32]
Healthcare	Healthcare expenditure	Access to healthcare		✓	[23, 24]
		Prompt treatment and available options		✓	
		Physician knowledge and H1N1awareness		✓	
Demographics	Population age structure	Preschool age	✓		[9]
		Pediatric patients	✓		[15]
		% < 15	✓		[16]
		% > 60	✓	✓	[16, 17]
		Children at home	✓		[34]
	Ethnicity	Alaskan/A.Indian/New Zealand aboriginal	✓		[71]
		Hipanics and blacks		✓	[13]
		Children of S. Asian descent	✓		[16]
pH1N1 Viral Factors	Strain variation by country	Start of the pandemic	✓		[30, 38, 39]
		Pandemic peak	✓		
		Co-circulation	✓	✓	
	Viral shedding/ transmission	Longer shedding in younger people	✓		[72]
		Longer shedding under optimal climatic conditions	✓		[26]
		Longer shedding in immunocompromised people	✓		[8, 72]
		Longer shedding or higher viral loads in those with severe disease	✓		
		Shorter shedding in those treated with antivirals within 48 h		✓	[8]
	Disease severity	Pathogenic strains	✓		
Data or modelling issues	Variation in case finding by country	-Clinician awareness		✓	
		-Diagnostic availability	✓	✓	
		-Testing protocol	✓	✓	
		-Breadth of surveillance	✓	✓	
Pandemic preparedness activities	Policies	Quarantine		✓	[35]
	Activities	Treatment, education		✓	
	Case finding efforts	Outreach and method	✓	✓	
	Quickness of response	Timing to mobilize community	✓	✓	
Other Factors	CDC Multiplier				[43]
	International flight traffic	International flights coming and going	✓		[36]

^a^ “Association Higher” indicates those factors that may be risk factors for mortality. “Association Lower” indicates those factors that may be protective against mortality. Some factors are found to be both risk factors and protective factors in the literature and this is indicated with two check marks in the table

Of the 48 possible risk factors identified, 27 factors passed the selection criteria listed above giving sufficient data to ensure optimized model calculations. Factors we were unable to explore due to insufficient data include comorbidities in children such as developmental delay, cerebral palsy, neurological disorders and congenital heart disease, adult immunosuppression other than HIV and TB, antiviral use within 48 h, pH1N1 strain variation by country, case finding methods, and pandemic preparedness activities. We identified data for 27 factors and in some cases the data for one factor acted as a proxy for some of the factors we couldn’t find sufficient data to explore. For example, we found data for health expenditure, which acts as a proxy for clinician awareness, quick access to treatment, and H1N1 knowledge, three factors we were unable to explore directly. The categories for the 27 factors, the direction of the believed association, the data measurement used in the model, the data sources and other details are listed in Table 2.

**Table 2:** The 27 factors selected from the literature search that were included in the modeling analysis

Factor type	Factor	Factor measurement for 2009	#	Metric unit	Data source	No. Countries with data	Value range or % – all countries	Value range or % – stage 1 countries	Stage 1 countries with data (20)
Environmental	Pollution	pm10	1	μg/m^3^	World Bank	167	6.7–156.2	11.7–60.2	✓
		co2 emissions	2	(KT) per 1000		174	0.06–6533.0	15.1–6533.0	✓
	Crowding	Pop. Density	3	Per square km		178	1.75–7125.1	2.9–7125.2	
	Cold Climate	Latitude	4	Absolute degree		178	0–65	1–56	✓
Physiological comorbidities in adults	Obesity	BMI > 30 (% both sexes)	5	%	WHO	176	1.1–59.6	4.5–33.5	
		BMI > 30 (% females)	6			175	19.8–36.3	21.7–29.9	✓
		BMI > 30 (% of males)30	7			175	20.1–32.7	22.7–29.3	
	Pulmonary Disease	Female COPD deaths	8	Per 100,000		173	4–125	7–89	✓
		Male COPD deaths	9			170	8–178	19–118	
	Immuno-suppression	%HIV	10	%	World Bank	145	0.1–25.9	0.1–17.8	✓
		TB	11	Per 100,00		172	0.8–1193	4.5–808	✓
		% HIV ARTs	12	%HIV * %HIV ARTS		102	0.004–22.3	0.07–7.5	
Physiological comorbidities in children	Asthma	%13 and 14 yr. olds with wheeze	13	%	Global Asthma Report	50	3–28	5.1–28	✓
Treatment	Antiviral Use	Kilos of drugs distributed 2007–2009	14	Per 100,000	IMS	60	0–16.2	0.06–15.1	
		Kilos of drugs distributed 2009	15			60	0–16.1	0.05–8.1	
Healthcare	Healthcare expenditure	Healthcare spending as %GDP	16	%	World Bank	173	2.0–16.2	3.9–16.2	
Demographics	Population age	% < 15	17	% total population	World Bank	176	13–50	13–31	✓
		% > 60	18			176	2–30	7–30	✓
	Population growth	Pregnancy -Crude birth rate	19	Per 1000		178	8.1–53.2	8.1–21.7	✓
pH1N1 Viral Factors	Pandemic factors	Pandemic Start	20	Weeks (1–52)	FluNet	81	5–46	5–37	✓
		Pandemic Peak	21			86	15–50	21–46	✓
	Pre and co-circulation	2008 H3N2 dominance	22	0 or 1 (1= > 50%)		69	34.8	35.3	
		2008 sH1N1 dominance	23			69	18.8	23.5	
		2009 H3N2 co-circulation	24	0 r 1 (1= > 1%)		90	46.7	52.6	
		2009 flu B co-circulation	25			90	35.6	21.1	✓
Other	CDC multiplier	Lower respiratory deaths	26	Per 100,000	CDC paper	169	3–275	11–107	✓
	International flight traffic	All registered take offs	27	Per 1000	World Bank	137	0–9182.4	25.5–9182.4	✓

### Risk factor influence on mortality

The results of the 16 model executions are presented in Table 3. The first four columns present the type of effect for each of the 16 models that were run for each variable: no effect (on influenza A(H1N1)pdm09 mortality), an association, a determinant effect, or a partial determinant effect. The numbers indicate how often a certain type of effect was found. Given our Decision Rules, it should be noted that an effect can be counted two times: a partial association and a partial determinant (see Additional file 1 - Technical Appendix), so the rows can add up to more then 16. The next two columns are counts of the direction of the relations that we found for these tests. The next column indicates our expected relation, based on our literature review, and the final column provides a measure of the impact the factor has on the difference between countries in excess mortality rates (captured in the model by the between country variance).

**Table 3:** Results of the 27 factors that were assessed in the 16 models

Factor components				Results					Effect direction		
Factor type	Factor	Factor measurement for 2009	#	No effect	Association	Determinant	Partial determinant	Country variance	Positive result	Negative result	Different than expected
Environmental	Pollution	pm10	1	10	3	5	2	4.03	6	0	
		co2 emissions	2	16	0	0	0	0.48	0	0	
	Crowding	Pop. density	3	12	2	2	0	1.38	4	0	
	Cold climate	Latitude	4	0	16	0	0	8.6	0	16	✓
Physiological comorbidities in adults	Obesity	BMI > 30 (% both sexes)	5	15	1	0	0	1	0	1	✓
		BMI > 30 (% of females)	6	14	1	1	0	0.61	1	1	✓
		BMI > 30 (% of males)	7	13	3	0	0	0.86	0	3	✓
	Pulmonary disease	Female COPD deaths	8	15	0	1	0	0.82	1	0	
		Male COPD deaths	9	12	2	2	0	1.57	4	0	
	Immuno-suppression	%HIV	10	7	5	4	0	4.26	9	0	
		%TB	11	11	2	3	0	2.05	5	0	
		% HIV ARTs	12	14	2	0	0	2.71	2	0	✓
Physiological comorbidities in children	Asthma	%13 and 14 yr. olds with wheeze	13	16	0	0	0	0.81	0	0	
Treatment	Antiviral use	Kilos of drugs distributed 2007–2009	14	15	0	1	0	2.21	0	1	
		Kilos of drugs distributed 2009	15	16	0	0	0	2.57	0	0	
Healthcare	Healthcare expenditure	Healthcare spending as %GDP	16	14	1	1	0	1.2	1	1	✓
Demographics	Population age	% < 15	17	0	16	2	2	35.2	16	0	
		% > 60	18	0	16	3	3	45.13	0	16	
	Population growth	Pregnancy -Crude birth rate	19	11	4	1	0	2.71	0	0	
pH1N1 Viral Factors	Pandemic factors	Pandemic Start	20	16	0	0	0	1.86	0	0	
		Pandemic Peak	21	15	1	0	0	2.76	0	1	✓
	Co-circulation	2008 H3N2 dominance	22	11	0	5	0	5.65	0	5	
		2008 sH1N1 dominance	23	16	0	0	0	1.62	0	0	
		2009 H3N2 co-circulation	24	7	0	9	0	8.08	9	0	
		2009 flu B co-circulation	25	12	0	4	0	2.99	4	0	
Other	CDC multiplier	Lower respiratory deaths	26	13	1	2	0	1.88	3	0	
	International flight traffic	All registered take offs	27	16	0	0	0	0.49	0	0	

All three aspects (type, direction, size of the effect) need to be combined to interpret the results. For example, one or two (partial) determinant effects together with almost no or a few associations probably indicates the factor did not play a significant role in explaining mortality differences, especially if the directions of the effects are different to what is expected (e.g. healthcare spending).

Of the 27 factors assessed, 9 showed a positive impact on mortality, either an association or as a determinant. Of the remaining factors, 10 had no effect in at least 15 out of the 16 model executions. The most consistent and strongest effects were found for the age structure of the population, explaining some 40% of the between country variance. Higher mortality was generally found in populations with a high percentage of persons aged <15 and lower mortalities were found in populations with an increased percentage of persons aged over 60, although these were more frequently associations. Considering age is a factor that is probably associated with several other variables that are related to excess mortality during the data generation process, it is hard (impossible) to create a dataset in which variation between countries due to the age distribution is absent. Latitude was another important variable (mean proportion explained variance of 7.8%), but there were mostly associations (rather than ‘determinant’ or ‘partial determinant’ effects) and most of the effect was probably the result of (low) mortality rates in Europe and the Northern Asian countries.

Other important determinant factors were the influenza types (A and B), both during the second half of 2009 and during the previous season, factors related to other infections (HIV, TB, lower respiratory deaths) and other respiratory stressors (pm10). No effect was found for healthcare spending or the distribution of antivirals. More detailed results for the different algorithms, observed samples and predictor sets are presented in the Additional file 1 - Technical Appendix together with the underlying regression models.

## Discussion

Our study investigates possible risk factors for influenza A H1N1 pandemic mortality in all world countries for 2009 using country data on pandemic respiratory mortality combined with global, standardized data on 27 possible risk factors identified in the literature. Our results illustrate that factors related to H1N1pdm severity and death are diverse, including previous and co-circulation of influenza subtypes, environmental factors, such as climate (latitude) and pollution, young age, and immunosuppression (HIV and TB). Latitude is probably reflective of the low mortality in Europe and Western Pacific rather than being important on its own. The other findings are similar in some respects to seasonal influenza, but different with respect to obesity [4, 7, 51], pregnancy [52, 53], and comorbidities such as asthma and COPD [2, 5, 6, 9] where we found no effect.

We found that the most important factor explaining why some countries were harder hit by influenza A(H1N1)pdm09 was age. Young age (% of the population < 15) contributes to 40% of the between country mortality variance. This result was not surprising as the frequency and severity of pH1N1 in younger ages compared to older age groups in seasonal influenza has been shown in numerous studies [15, 18, 19].

We also found a prominent mortality impact from influenza subtypes H3N2 and Influenza B when co-circulating with pH1N1 in 2009 and a protective effect in countries where H3N2 dominated in 2008. These findings suggest that a population’s immunological response to a virus in the previous or current season (due to factors like waning immunity or the original antigenic sin) had an impact on mortality levels in 2009.Vaccine effectiveness studies have shown that immunological responses need to be considered when explaining findings, with persons who were vaccinated in the previous season having lower vaccine effectiveness rates compared to those who were not vaccinated during the previous season [37, 54, 55].

Although our study was unable to investigate all comorbidities believed to impact influenza mortality, we did find a strong determinant effect with immune-compromising chronic infections (HIV prevalence and a smaller effect for TB) [12, 56, 57]. Finally, we found that environmental exposures are a risk factor if they are a burden for the respiratory system e.g. air quality-CO2 and pm10 [28].

We found no association between pandemic mortality in 2009 and factors such as antiviral stockpiling [31, 58, 59], medical and economic factors (e.g. health expenditure) [23, 24], international air travel [36], previous circulation of seasonal H1N1 [37], or the pandemic timing in a country – the start and peak [27, 30]. We were not able to analyze all factors and additional factors that would remain worthy of investigation, include viral and bacterial co-infection [12], use of antivirals within 48 h [9, 29, 33, 60], preexisting influenza immunity [61], diabetes, neurological disorders [11, 16], the availability and use of advanced treatment options such as ECMO and pandemic preparedness activities and policies [30, 35].

### Modelling strategy

Different regression strategies are conceivable to establish a relation between the risk factors and the estimated excess pandemic mortality rates. A first strategy would be to base the regression analysis on the 20 observed countries included in the GLaMOR project for which we calculated a country-specific pandemic mortality estimate based on a standardized approach [41]. However, it is well known from the statistical literature that such a small sample makes the analysis vulnerable to numerous problems and has significant limitations [62]. For completeness, we have performed these regressions and present the results in the appendix (see Table C).

A second strategy, using traditional regression procedures, would relate the country specific excess rates (20 + 170) estimated using the GLaMOR approach [41] to a risk factor (or a group of risk factors). This strategy has two major problems: 1.) If the factor being investigated was used in the GLaMOR data creation step (there were 10 “factors” used in the GLaMOR study) it will very often give a false positive relation; 2.) If the factor being investigated isn’t present in the GLaMOR data simulation exercise then it will probably give a false negative relation (unless the relation is introduced via another predictor in the GLaMOR procedure). Therefore, doing a traditional regression analysis using the GLaMOR data combined with the identified risk factors variables will lead to biased results with limited meaning. The most limiting factor of both strategies is that the nature of an association (association or determinant effect) cannot be distinguished, a well-known limitation of regression analysis. To address these different points, we chose a modeling strategy which uses data simulations and regression models and is based on the Rubin’s Causal Inference Model [48].

### Limitations of our study include

- Inability to study the pandemic in 2010 and it is known that there was more than one wave with varying impacts.
- Inability to explore some important factors due to insufficient data including antiviral use within 48 h (we only had sales), strain pathogenicity, viral shedding, co-morbidities in children such as neurological disorders, morbid obesity, and pandemic preparedness activities and approaches.
- The validity and comparability of the global datasets. Some of the 27 factors that were included in our modeling procedure use global datasets that are not complete (i.e. do not cover all countries in the world, see Table 2) or accurate. For example, we used the WHO FluNet database for the ‘Co-circulation viruses’ factors but the validity and comparability of this dataset has been questioned by a global spatiotemporal study [63], with the study finding striking differences in the proportion of influenza B cases across America, Europe and Asia which may be explained by different testing efforts across countries / regions.
- Some of the factors we investigated were intended to be a proxy for something else. For example, latitude was a proxy for climate and birth rate was a proxy for pregnancy and for the number of children in the household. In addition, there is the possibility of misinterpretation of our results due to the influence of age. Countries close to the equator have a large population of young people compared to countries in the North. Our data on circulating strains of influenza during and prior to the pandemic could be strengthened with more country data to be entered during the data creation steps of the model
- Although this study gives a clear picture of factors that created variation in the pandemic excess mortality between countries, the results should be confirmed in new studies. Some of the factors were measured in a very approximate manner, for instance, the antivirals are based on distribution data rather than a measure of actual use. Or the influenza virus surveillance data was only available for a limited number of countries and the representativeness of this data was unclear (was it a good sample of what is going on in a county and are they comparable between years and countries).

## Conclusion

The story of pandemic mortality burden variations in 2009 has not been clear. Mostly age, pregnancy, obesity, and co-morbidities have been used to explain observed differences, although even these factors continue to be disputed in the literature [14, 40, 64–68]. Although our study attempts to simplify the complexity of this topic, it provides some clarity but also raises some further areas for analysis. We reconfirm the association of age with mortality, finding higher mortality in lower age groups [15, 18, 19]. We also highlight the need to look at environmental exposures which are a burden on the respiratory system (e.g. air pollution) immune-compromising chronic infections (e.g. HIV and TB), and the interference of other influenza types co-circulating or previously circulating which appear to change the risk of mortality [41].

Possibly the most important result is the relation between variation in immune response reactions [61], based on viruses that circulated in previous seasons or that co-circulated with the 2009 pandemic virus, and variations in the mortality impact. Considering individuals have different immune responses to influenza virus infections over time (including following vaccination), this could explain the age effect of the 2009 pandemic, when a population was confronted with a new influenza virus (ignoring the environment and history). Our findings suggest that individuals who always generate a less optimal immune response to a (specific) influenza infection had died during previous influenza seasons and among the elderly this group would be smaller than among younger individuals. Also, the older had been exposed to more frequent virus infections [61], including influenza AH1N1 viruses, and this may have protected this group from the 2009 pandemic virus. In conclusion, besides general differences in individual responses to influenza viruses, our study finds that the context (previous and co-circulation of different types of influenza virus, other bacterial and viral activity) plays an important role in defining whether an influenza virus infection results in death during a pandemic.

These findings have important implications for research and public health actions. Variation in immune responses to the same infection should be studied not only in general but also within different contexts (previous and co-circulation of different types of influenza virus, other bacterial and viral activity). Our study suggests that vaccine studies should assess the impact of previous influenza infections (other influenza types), as in a worst case scenario a vaccine could, potentially, increase the chance of mortality, both at an individual and at a population level. The findings are probably also relevant to seasonal influenza epidemics and it would be useful to repeat this analysis on data for the seasonal mortality impact of influenza.

Interestingly, we found no effect for some factors long believed to matter, which has implications for future pandemic planning and response. These factors include obesity [29, 40], pregnancy [19, 22, 40, 52, 69] and stockpiling of antivirals [31, 58, 70]. This result does not dismiss those risk factors entirely, but should draw attention to the possibility of identifying false risk factors through the current practice of monitoring an epidemic (in real time and at the earliest stages).

In conclusion, besides general differences in individual responses to influenza viruses, our study finds that the context (previous and co-circulation of different types of influenza virus, other bacterial and viral activity) plays an important role in defining whether an influenza virus infection results in death during a pandemic. We also show that identifying mortality risk factors in a pandemic is a complex exercise and that real time assessments of the 2009 pandemic, frequently based on data from one country, probably led to many false positive findings. Finally, we feel that in the future more sophisticated (statistical) methods using data from multiple countries, preferably from different continents, should be used to assess the risk factors of pandemic mortality.

## Additional file

Additional file 1: Supporting Information: Technical Appendix. (DOC 1315 kb)

## Abbreviations

ART: Anti-retroviral therapy
BMI: Body Mass Index
CDC: Centers for Disease Control and Prevention
CO2: Carbon dioxide
COPD: Chronic Obstructive Pulmonary Disease
ECMO: Extracorporeal membrane oxygenation
GLaMOR: Global Pandemic Mortality
HIV: Human immunodeficiency virus
KT: kiloton
pm: particulate matter
TB: Tuberculosis
UN: United Nations
WHO: World Health Organization
