# Supplementary material for: Possible explanations for why some countries were harder hit by the pandemic influenza virus in 2009 – a global mortality impact modeling study
Source: BMC Infect Dis. 2017 Sep 25;17:642. doi: 10.1186/s12879-017-2730-0 (PMC5613504; doi:10.1186/s12879-017-2730-0)
Supplement: Additional file 1: — Supporting Information: Technical Appendix. (DOC 1315 kb) [file 12879_2017_2730_MOESM1_ESM.doc]

**Supporting Information: Technical Appendix**

In this Appendix we will provide more information regarding the methodology and analysis strategy. The overall procedure consists of four elements.

First, there is the observed sample(s) of excess mortality rates. We call them observed as it is based on real measurements done in 20 countries. For a more elaborate discussion of this methodology, see the Simonsen et al paper [41]. Second are the predictor sets, of which there are four sets (see Table 1 below), and third there are the algorithms used (the matching and imputation algorithms). By using these different elements we were able to produce 16 plausible excess mortality rates per country (4 predictors sets x 2 algorithms x 2 age groups (<65 and 65+)).

The most important difference between the two algorithms is the way they use the available information. Imputations use all available information simultaneously, the sample of the observed excess mortality together with all the variables in the predictor set and the relations amongst them, to create a series of plausible values for the countries with missing excess mortality using statistical assumptions on the distribution of these values (the imputation model). Matching takes a different approach and matches the observed excess mortality rates to neighboring countries based on only one predictor variable (see the Simonsen et al paper [41], in particular the Appendix, for a detailed description [41]) . This procedure is repeated for each of the predictor variables, to create a dataset for that country of values of excess mortality. The matching algorithm ignores the relation between all the variables and makes minimal assumptions about these relations. Imputation can be viewed as a parametric algorithm and matching as a non-parametric algorithm.

The fourth element of the calculation procedure is a hierarchical linear model which integrates these different plausible values into one excess mortality rate per country, including a measure of its uncertainty, while controlling for potential measurement error in the different data sets.

The imputations and matching procedures were done with the Amelia (R package) software [74] and the hierarchical linear model was done with MLwiN 2.30 [75].

***Table 1 - Predictor Sets Used***

*Data come from the UN (data.un.org), WHO (www.who.int/gho/en), WORLDBANK (data.worldbank.org)*

| **PREDICTOR SETS USED** | | | | | |
| --- | --- | --- | --- | --- | --- |
|  | **PREDICTOR SET 1** | | **PREDICTOR SET 2** | **PREDICTOR SET 3** | **PREDICTOR SET 4** |
| **DATA INCLUDED** | WHO regions (6) | WHO regions (6) | | WHO regions (6) | WHO regions (6) |
|  | Deaths per 1000, by age group (0-14, 15-59, 65+), 2005-2010 | Deaths per 1000, by age group (0-14, 15-59, 65+), 2005-2010 | | Deaths per 1000, by age group (0-14, 15-59, 65+), 2005-2010 |  |
|  | Physician density |  | | Physician density | Physician density |
|  | Obese % population bmi>30 | Obese % population bmi>30 | | Obese % population bmi>30 |  |
|  | population density |  | | population density | population density |
|  | HIV, total (% of population ages 15-49) | HIV, total (% of population ages 15-49) | | HIV, total (% of population ages 15-49) |  |
|  | Tuberculosis (per 100 000) | Tuberculosis (per 100 000) | | Tuberculosis (per 100 000) |  |
|  | GNI per capita, PPP ( $) | GNI per capita, PPP ( $) | | GNI per capita, PPP ( $) | GNI per capita, PPP ( $) |
|  | Rural population % |  | | Rural population % | Rural population % |
|  | Population aged under 15 (%) | Population aged under 15 (%) | | Population aged under 15 (%) | Population aged under 15 (%) |
|  | Population aged over 60 (%) | Population aged over 60 (%) | | Population aged over 60 (%) | Population aged over 60 (%) |
|  | Latitude (absolute value) | Latitude (absolute value) | | Latitude (absolute value) | Latitude (absolute value) |
|  |  | MRI machines per 1 000 000 | | MRI machines per 1 000 000 | MRI machines per 1 000 000 |
|  |  | Life expectancy | | Life expectancy |  |
|  |  | Crude Birth rate (per 1,000 people) | | Crude Birth rate (per 1,000 people) |  |
|  |  | Health expenditure (% of GDP) | | Health expenditure (% of GDP) | Health expenditure (% of GDP) |
|  |  | Cardio and diabetes deaths per 100,000 | | Cardio and diabetes deaths per 100,000 |  |
|  |  | Cancers, deaths per 100,000 | | Cancers, deaths per 100,000 | Cancers, deaths per 100,000 |
|  |  | Chronic respiratory deaths per 100,000 | | Chronic respiratory deaths per 100,000 |  |
|  |  | Hospital bed density (per 10,000) | | Hospital bed density (per 10,000) | Hospital bed density (per 10,000) |
|  |  |  | | Employ % population | Employ % population |
|  |  |  | | CO2 emission | CO2 emission |
|  |  |  | | Energy use | Energy use |
|  |  |  | | Food product index | Food product index |
|  |  |  | | Gross savings | Gross savings |
|  |  |  | | Internet use | Internet use |
|  |  |  | | mean years schooling people over 25 | mean years schooling people over 25 |
|  |  |  | | Maternal mortality |  |
|  |  |  | | Mobile phones | Mobile phones |
|  |  |  | | Population growth | Population growth |
|  |  |  | | Primary educ complete | Primary educ complete |
|  |  |  | | Public spending education | Public spending education |
|  |  |  | | Human development index | Human development index |
|  |  |  | | Refugees | Refugees |
|  |  |  | | Tax revenue % of gdp | Tax revenue % of gdp |
|  |  |  | | Expected years of schooling | Expected years of schooling |
|  |  |  | | Air transport | Air transport |
| **DESCRIPTION** | Same predictor set used in the PLoS Med article by Simonson, et al. | BASIC | | BASIC+EXTRA | (BASIC+EXTRA) but removing all WHO_related variables |
| **RESULTS TABLES GENERATED** | B1,B2,B9,B10 | B3,B4,B11,B12 | | B5,B6,B13,B14 | B7,B8,B15,B16 |

The model is a slight adaptation from the model that was used in the Simonsen et al paper [41]. The regional component is not needed and the factor of interest is added and sometimes the age (%<15, %>60) confounder is added (see below Decision Rules below).


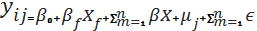


Y = excess mortality rate

i = measurement

j = country


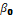
 = intercept


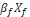
= factor of interest and age confounder(fixed effect)


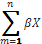
= indicators(m) for matched or imputed datasets (fixed effect)

coding ((0,1)-1/n)

n=number of datasets


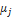
 = between country variance (random effect)


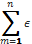
 = error variance for each dataset

coding (0,1)

**Decision rules to establish the nature of the effect**

We ran the following procedure for each factor that we assessed.

In one model the factor is absent from the predictor set and the plausible values are created; next a bivariate regression is run based on the two datasets (the factor versus the estimated pandemic mortality_0_). The null hypothesis (H(0)) that is being tested is that there is no relation between the factor and excess pandemic mortality.

In the second model the factor is present in the predictor set and the plausible values are created; next a bivariate regression based on the two datasets is run (the factor versus the estimated pandemic mortality_2_). The hypothesis that is being tested here is that there is a relation (the alternative hypothesis H(1)) between the factor and excess pandemic mortality.

To establish the nature of the effect of a variable on pandemic mortality, we first we have to decide when to call a regression coefficient significant. This is done in the usual manner by calculating the t-value (estimate/standard error) and looking up the associated p-value. If this is smaller than 0.05 we consider the effect as significant.

Considering it is possible that in both situations (under H0 and H1) a significant effect is found, we also tested whether the difference between the two regression coefficients was significant. If we have a specific expectation about the relation between the factor and the excess mortality, we performed a one-sided test for the difference (for instance absent (H0) effect smaller than the present (H1) effect).

Impact of age structure on our analysis:

A particular problem with our data is that we know that the mortality risk is age dependent (more young people died then elderly) [41]. And since the population age structure in a country (percentage under 15 years, and percentage above 60 year) is present in all the 4 predictor sets, we expect many association effects to occur because it probably wrongly takes over the age effect (without the age effect there would have no effect on its own). This false effect under both situations is the result of the correlation between the factor and the age structure. Age therefore creates the variability in the excess mortality and the regression establishes the (false) relation with this other factor. To address this issue, the regressions are re-run with the two age confounders present to control for these artifacts.

To assess these different factors, the following Decision Rules were implemented for our analysis. ‘Stop’ means the type of effect is identified, ‘Absent’ means the situation under H0 and ‘Present’ means the situation under H1

- Absent: No effect & Present: no effect -> No effect (Stop)

- Absent: No effect & Present: an effect & the sign of the correlation is in the expected direction -> Determinant effect (Stop)

- All other situations -> first re-run the regression with confounders to adjust for age:

- Absent: No effect & Present: no effect -> No effect (Stop)

- Absent: No effect & Present: effect& sign in the correlation in the expected direction -> Determinant effect (Stop)

- Absent: effect & Present: no effect-> No effect (Stop)

- Absent: No effect & Present: effect & sign in the correlation in an unexpected direction -> Association effect (Stop)

- Absent: Effect & Present: effect & difference effect & no expected direction-> Association effect (Stop)

- Absent: Effect & Present: effect & difference effect & sign in unexpected direction->Association effect (Stop)

- Absent: Effect & Present: effect & difference effect & sign in expected direction-> Partial Determinant effect and Partial association effect (Stop)

This covers all of the situations we could have encountered. Note: we call an effect a determinant effect only if a difference is created in the data generation process in an expected direction. This means that effects classified as Association can have the potential of being a Determinant but that we couldn’t make the distinction with our procedure. Another important point is that falsification of the determinant effect is harder than verification using this procedure.

**References:**

[74] Honaker K. G, Blackwell. Amelia II: a program for missing data. J Stat Softw. 2011;45(7):1–47.

[75] Rasbash J, Charlton C, Browne WJ, Healy M, Cameron B. MLwiN version 2.1. Centre for Multilevel Modelling, university of Bristol, 2009.

**Additional results**

**Tables A (1-2) - Complete effects tables**

***Table A1 – All Age***

| **ALL AGES TABLE A** | **PREDICTOR SET 1** | | **PREDICTOR SET 2** | | **PREDICTOR SET 3** | | **PREDICTOR SET 4** | |
| --- | --- | --- | --- | --- | --- | --- | --- | --- |
| **FACTOR NAMES** | **MATCHING** | **IMPUTATION** | **MATCHING** | **IMPUTATION** | **MATCHING** | **IMPUTATION** | **MATCHING** | **IMPUTATION** |
| Pm10 | **C** | **D** | **A** | **D** | **A** | **C** | **B** | **A** |
| Co2 emissions | **A** | **A** | **A** | **A** | **A** | **A** | **A** | **A** |
| Pop. density | **A** | **C** | **B** | **A** | **B** | **A** | **A** | **C** |
| Lattitude | **B** | **B** | **B** | **B** | **B** | **B** | **B** | **B** |
| % BMI >30 | **A** | **B** | **A** | **A** | **A** | **A** | **A** | **A** |
| % female BMI >30 | **A** | **B** | **A** | **A** | **A** | **A** | **A** | **A** |
| % male BMI >30 | **A** | **B** | **A** | **A** | **A** | **A** | **A** | **A** |
| Female COPD deaths | **A** | **C** | **A** | **A** | **A** | **A** | **A** | **A** |
| Male COPD deaths | **C** | **A** | **A** | **A** | **A** | **A** | **A** | **A** |
| % HIV | **C** | **C** | **A** | **A** | **A** | **B** | **A** | **A** |
| % TB | **A** | **C** | **A** | **A** | **A** | **A** | **A** | **C** |
| % HIV ARTs | **A** | **A** | **A** | **A** | **A** | **A** | **A** | **A** |
| % 13-14 yr olds with wheeze | **A** | **A** | **A** | **A** | **A** | **A** | **A** | **A** |
| Kilos of drugs distr. 2007-2009 | **A** | **A** | **A** | **A** | **A** | **A** | **A** | **A** |
| Kilos of drugs distr. 2009 | **A** | **A** | **A** | **A** | **A** | **A** | **A** | **A** |
| Health care spending as % GDP | **A** | **A** | **A** | **A** | **A** | **A** | **A** | **A** |
| % <15 | **B** | **B** | **B** | **B** | **B** | **D** | **B** | **B** |
| % > 60 | **B** | **B** | **B** | **B** | **B** | **D** | **B** | **D** |
| Crude birth rate | **A** | **A** | **A** | **B** | **A** | **C** | **A** | **A** |
| Pandemic Start | **A** | **A** | **A** | **A** | **A** | **A** | **A** | **A** |
| Pandemic Peak | **B** | **A** | **A** | **A** | **A** | **A** | **A** | **A** |
| 2008 H3N2 dominance | **C** | **C** | **A** | **A** | **A** | **C** | **C** | **C** |
| 2008 sH1N1 dominance | **A** | **A** | **A** | **A** | **A** | **A** | **A** | **A** |
| 2009 H3N2 co-circulation | **C** | **C** | **C** | **C** | **C** | **C** | **C** | **C** |
| 2009 flu B co-circulation | **A** | **A** | **C** | **C** | **A** | **A** | **A** | **A** |
| Lower respiratory deaths | **A** | **C** | **A** | **A** | **A** | **A** | **A** | **A** |
| All registered take offs | **A** | **A** | **A** | **A** | **A** | **A** | **A** | **A** |

***A = NO EFFECT***

***B = ASSOCIATION***

***C = DETERMINANT***

***D = PARTIAL-DETERMINANT***

Table A2 - <65 Age group

| **<65 AGES TABLE A** | **PREDICTOR SET 1** | | **PREDICTOR SET 2** | | **PREDICTOR SET 3** | | **PREDICTOR SET 4** | |
| --- | --- | --- | --- | --- | --- | --- | --- | --- |
| **FACTOR NAMES** | **MATCHING** | **IMPUTATION** | **MATCHING** | **IMPUTATION** | **MATCHING** | **IMPUTATION** | **MATCHING** | **IMPUTATION** |
| Pm10 | A | A | A | C | A | A | A | A |
| Co2 emissions | A | A | A | A | A | A | A | A |
| Pop. density | A | A | A | A | A | A | A | A |
| Lattitude | B | B | B | B | B | B | B | B |
| % BMI >30 | A | A | A | A | A | A | A | A |
| % female BMI >30 | A | A | A | A | A | A | C | A |
| % male BMI >30 | A | A | A | B | A | B | A | A |
| Female COPD deaths | A | A | A | A | A | A | A | A |
| Male COPD deaths | A | A | B | A | A | B | C | A |
| % HIV | B | A | B | A | B | B | C | C |
| % TB | A | A | A | B | A | B | A | C |
| % HIV ARTs | A | A | B | A | A | B | A | A |
| % 13-14 yr olds with wheeze | A | A | A | A | A | A | A | A |
| Kilos of drugs distr. 2007-2009 | A | A | A | A | A | C | A | A |
| Kilos of drugs distr. 2009 | A | A | A | A | A | A | A | A |
| Health care spending as % GDP | B | C | A | A | A | A | A | A |
| % <15 | B | B | B | D | B | B | B | B |
| % > 60 | B | B | B | B | B | D | B | B |
| Crude birth rate | B | A | A | B | A | B | A | A |
| Pandemic Start | A | A | A | A | A | A | A | A |
| Pandemic Peak | A | A | A | A | A | A | A | A |
| 2008 H3N2 dominance | A | A | A | A | A | A | A | A |
| 2008 sH1N1 dominance | A | A | A | A | A | A | A | A |
| 2009 H3N2 co-circulation | A | A | A | A | A | A | A | C |
| 2009 flu B co-circulation | A | A | C | A | A | A | C | A |
| Lower respiratory deaths | A | A | A | B | A | C | A | A |
| All registered take offs | A | A | A | A | A | A | A | A |

***A = NO EFFECT***

***B = ASSOCIATION***

***C = DETERMINANT***

***D = PARTIAL-DETERMINANT***

**Tables B (1-16) - Regression results tables**

***Table B1 – All Age, Predictor Set 1, Matching***

| **FACTOR NAMES** | **REGRESSION COEFFICIENT** | **P-VALUE UNDER H(0)** | **REGRESSION COEFFICIENT** | **P-VALUE UNDER H(1)** | **PROPORTION (%) BETWEEN COUNTRY VARIANCE** **REDUCTION** |
| --- | --- | --- | --- | --- | --- |
| Pm10 | 0.0075 | 0.0001 | 0.0081 | 0.0000 | 3.31 |
| Co2 emissions | 0.0000 | 0.7413 | 0.0000 | 0.7936 | 0.03 |
| Pop. density | 0.0002 | 0.0074 | 0.0002 | 0.0070 | -0.12 |
| Lattitude | -0.0208 | 0.0000 | -0.0215 | 0.0000 | 16.09 |
| % BMI >30 | -0.0135 | 0.0050 | -0.0119 | 0.0118 | 0.03 |
| % female BMI >30 | -0.0168 | 0.4103 | -0.0109 | 0.5816 | 0.12 |
| % male BMI >30 | -0.1040 | 0.0000 | -0.0888 | 0.0000 | 0.07 |
| Female COPD deaths | 0.0146 | 0.0000 | 0.0155 | 0.0000 | 0.82 |
| Male COPD deaths | 0.0088 | 0.0000 | 0.0091 | 0.0000 | 3.19 |
| % HIV | 0.0493 | 0.0004 | 0.0559 | 0.0000 | 4.59 |
| % TB | 0.0012 | 0.0000 | 0.0012 | 0.0000 | 0.92 |
| % HIV ARTs | 0.0240 | 0.1242 | 0.0247 | 0.1176 | 2.98 |
| % 13-14 yr olds with wheeze | 0.0069 | 0.7687 | 0.0066 | 0.7735 | 0.23 |
| Kilos of drugs distr. 2007-2009 | -0.0178 | 0.5691 | -0.0169 | 0.5842 | 0.52 |
| Kilos of drugs distr. 2009 | -0.0509 | 0.2559 | -0.0465 | 0.3005 | 1.85 |
| Health care spending as % GDP | -0.0612 | 0.0012 | -0.0617 | 0.0011 | 1.51 |
| % <15 | 0.0382 | 0.0000 | 0.0404 | 0.0000 | 42.06 |
| % > 60 | -0.0666 | 0.0000 | -0.0705 | 0.0000 | 56.83 |
| Crude birth rate | 0.0380 | 0.0000 | 0.0373 | 0.0000 | 1.30 |
| Pandemic Start | -0.0156 | 0.1531 | -0.0178 | 0.0639 | 4.79 |
| Pandemic Peak | -0.0276 | 0.0018 | -0.0289 | 0.0009 | 5.93 |
| 2008 H3N2 dominance | -0.5772 | 0.0007 | -0.6394 | 0.0000 | 17.99 |
| 2008 sH1N1 dominance | 0.2101 | 0.3451 | 0.2371 | 0.1392 | 3.76 |
| 2009 H3N2 co-circulation | 0.5789 | 0.0000 | 0.6188 | 0.0000 | 19.35 |
| 2009 flu B co-circulation | 0.4233 | 0.0057 | 0.3829 | 0.0004 | 3.65 |
| Lower respiratory deaths | 0.0044 | 0.0000 | 0.0046 | 0.0000 | 2.56 |
| All registered take offs | 0.0000 | 0.6417 | 0.0000 | 0.5899 | 0.28 |

***Table B2 – All Age, Predictor Set 1,*** Imputation

| **FACTOR NAMES** | **REGRESSION COEFFICIENT** | **P-VALUE UNDER H(0)** | **REGRESSION COEFFICIENT** | **P-VALUE UNDER H(1)** | **PROPORTION (%) BETWEEN COUNTRY VARIANCE** **REDUCTION** |
| --- | --- | --- | --- | --- | --- |
| Pm10 | 0.0151 | 0.0000 | 0.0228 | 0.0000 | 20.65 |
| Co2 emissions | -0.0001 | 0.4569 | -0.0001 | 0.3274 | 0.57 |
| Pop. density | 0.0002 | 0.1332 | 0.0002 | 0.0342 | 2.60 |
| Lattitude | -0.0361 | 0.0000 | -0.0376 | 0.0000 | 12.71 |
| % BMI >30 | -0.0253 | 0.0008 | -0.0267 | 0.0002 | 2.43 |
| % female BMI >30 | -0.0344 | 0.2910 | -0.1004 | 0.0034 | 2.48 |
| % male BMI >30 | -0.1674 | 0.0000 | -0.2159 | 0.0000 | 3.57 |
| Female COPD deaths | 0.0205 | 0.0000 | 0.0280 | 0.0000 | 3.61 |
| Male COPD deaths | 0.0116 | 0.0000 | 0.0146 | 0.0000 | 1.72 |
| % HIV | 0.0267 | 0.1917 | 0.0505 | 0.0120 | 4.44 |
| % TB | 0.0014 | 0.0001 | 0.0017 | 0.0000 | 3.38 |
| % HIV ARTs | -0.0204 | 0.3808 | -0.0359 | 0.1338 | 2.41 |
| % 13-14 yr olds with wheeze | -0.0065 | 0.8459 | -0.0198 | 0.5244 | 0.82 |
| Kilos of drugs distr. 2007-2009 | -0.0507 | 0.2962 | -0.0366 | 0.4085 | 1.17 |
| Kilos of drugs distr. 2009 | -0.1017 | 0.1441 | -0.0856 | 0.2157 | 2.53 |
| Health care spending as % GDP | -0.0990 | 0.0018 | -0.1300 | 0.0000 | 2.33 |
| % <15 | 0.0457 | 0.0000 | 0.0581 | 0.0000 | 29.33 |
| % > 60 | -0.0995 | 0.0000 | -0.0783 | 0.0000 | 29.52 |
| Crude birth rate | 0.0522 | 0.0000 | 0.0663 | 0.0000 | 1.00 |
| Pandemic Start | -0.0150 | 0.4157 | -0.0273 | 0.0869 | 3.68 |
| Pandemic Peak | -0.0366 | 0.0146 | -0.0315 | 0.0453 | 0.73 |
| 2008 H3N2 dominance | -0.9614 | 0.0006 | -1.0751 | 0.0000 | 9.39 |
| 2008 sH1N1 dominance | 0.0942 | 0.7976 | -0.4484 | 0.2751 | 1.73 |
| 2009 H3N2 co-circulation | 0.8987 | 0.0001 | 1.4491 | 0.0000 | 17.40 |
| 2009 flu B co-circulation | 0.6361 | 0.0133 | 0.3944 | 0.0687 | 3.77 |
| Lower respiratory deaths | 0.0065 | 0.0001 | 0.0098 | 0.0000 | 6.19 |
| All registered take offs | 0.0000 | 0.7230 | -0.0001 | 0.5641 | 0.25 |

***Table B3 – All Age, Predictor Set 2,*** Matching

| **FACTOR NAMES** | **REGRESSION COEFFICIENT** | **P-VALUE UNDER H(0)** | **REGRESSION COEFFICIENT** | **P-VALUE UNDER H(1)** | **PROPORTION (%) BETWEEN COUNTRY VARIANCE** **REDUCTION** |
| --- | --- | --- | --- | --- | --- |
| Pm10 | 0.0060 | 0.0006 | 0.0064 | 0.0003 | 1.59 |
| Co2 emissions | 0.0000 | 0.8209 | 0.0000 | 0.8485 | 0.02 |
| Pop. density | 0.0002 | 0.0005 | 0.0002 | 0.0006 | 2.74 |
| Lattitude | -0.0174 | 0.0000 | -0.0178 | 0.0000 | 8.31 |
| % BMI >30 | -0.0070 | 0.1042 | -0.0066 | 0.1274 | 1.40 |
| % female BMI >30 | -0.0037 | 0.8409 | -0.0016 | 0.9301 | 0.00 |
| % male BMI >30 | -0.0764 | 0.0002 | -0.0705 | 0.0005 | 0.15 |
| Female COPD deaths | 0.0118 | 0.0000 | 0.0124 | 0.0000 | 0.42 |
| Male COPD deaths | 0.0068 | 0.0000 | 0.0070 | 0.0000 | 1.08 |
| % HIV | 0.0438 | 0.0006 | 0.0468 | 0.0001 | 2.88 |
| % TB | 0.0009 | 0.0000 | 0.0009 | 0.0000 | 0.08 |
| % HIV ARTs | 0.0198 | 0.1666 | 0.0200 | 0.1613 | 2.18 |
| % 13-14 yr olds with wheeze | -0.0137 | 0.5641 | -0.0138 | 0.5572 | 0.72 |
| Kilos of drugs distr. 2007-2009 | -0.0196 | 0.5326 | -0.0188 | 0.5481 | 0.61 |
| Kilos of drugs distr. 2009 | -0.0525 | 0.2433 | -0.0492 | 0.2777 | 1.99 |
| Health care spending as % GDP | -0.0615 | 0.0003 | -0.0618 | 0.0003 | 0.11 |
| % <15 | 0.0309 | 0.0000 | 0.0322 | 0.0000 | 30.10 |
| % > 60 | -0.0569 | 0.0000 | -0.0591 | 0.0000 | 43.93 |
| Crude birth rate | 0.0276 | 0.0000 | 0.0276 | 0.0000 | 0.03 |
| Pandemic Start | -0.0115 | 0.2756 | -0.0130 | 0.1916 | 2.22 |
| Pandemic Peak | -0.0160 | 0.0653 | -0.0168 | 0.0508 | 4.47 |
| 2008 H3N2 dominance | -0.5177 | 0.0016 | -0.5629 | 0.0001 | 5.88 |
| 2008 sH1N1 dominance | 0.2037 | 0.3378 | 0.2203 | 0.2236 | 2.33 |
| 2009 H3N2 co-circulation | 0.5493 | 0.0000 | 0.5816 | 0.0000 | 6.81 |
| 2009 flu B co-circulation | 0.4982 | 0.0004 | 0.4723 | 0.0001 | 6.06 |
| Lower respiratory deaths | 0.0029 | 0.0030 | 0.0030 | 0.0020 | 0.16 |
| All registered take offs | 0.0000 | 0.9247 | 0.0000 | 0.8895 | 0.02 |

***Table B4 – All Age, Predictor Set 2,*** Imputation

| **FACTOR NAMES** | **REGRESSION COEFFICIENT** | **P-VALUE UNDER H(0)** | **REGRESSION COEFFICIENT** | **P-VALUE UNDER H(1)** | **PROPORTION (%) BETWEEN COUNTRY VARIANCE** **REDUCTION** |
| --- | --- | --- | --- | --- | --- |
| Pm10 | 0.0168 | 0.0000 | 0.0251 | 0.0000 | 14.31 |
| Co2 emissions | -0.0002 | 0.1945 | -0.0002 | 0.1568 | 1.22 |
| Pop. density | 0.0001 | 0.3459 | 0.0001 | 0.5125 | 0.26 |
| Lattitude | -0.0328 | 0.0000 | -0.0403 | 0.0000 | 6.23 |
| % BMI >30 | -0.0213 | 0.0169 | -0.0392 | 0.0001 | 0.61 |
| % female BMI >30 | -0.0267 | 0.4816 | -0.0291 | 0.4736 | 0.31 |
| % male BMI >30 | -0.2067 | 0.0000 | -0.2084 | 0.0000 | 0.14 |
| Female COPD deaths | 0.0309 | 0.0000 | 0.0277 | 0.0000 | 0.72 |
| Male COPD deaths | 0.0133 | 0.0000 | 0.0183 | 0.0000 | 0.01 |
| % HIV | 0.1201 | 0.0000 | 0.0986 | 0.0003 | 0.74 |
| % TB | 0.0025 | 0.0000 | 0.0028 | 0.0000 | 0.56 |
| % HIV ARTs | 0.0856 | 0.0149 | 0.0296 | 0.3660 | 0.86 |
| % 13-14 yr olds with wheeze | -0.0079 | 0.8087 | -0.0175 | 0.6050 | 0.55 |
| Kilos of drugs distr. 2007-2009 | -0.0504 | 0.2589 | -0.0469 | 0.3410 | 1.52 |
| Kilos of drugs distr. 2009 | -0.1015 | 0.1125 | -0.0547 | 0.4674 | 0.89 |
| Health care spending as % GDP | -0.1810 | 0.0000 | -0.2410 | 0.0000 | 1.74 |
| % <15 | 0.0940 | 0.0000 | 0.0838 | 0.0000 | 42.09 |
| % > 60 | -0.1603 | 0.0000 | -0.1666 | 0.0000 | 61.53 |
| Crude birth rate | 0.0831 | 0.0000 | 0.0850 | 0.0000 | 4.89 |
| Pandemic Start | -0.0033 | 0.8541 | -0.0273 | 0.1330 | 2.83 |
| Pandemic Peak | -0.0233 | 0.1306 | -0.0288 | 0.0805 | 3.53 |
| 2008 H3N2 dominance | -0.9315 | 0.0016 | -1.1280 | 0.0007 | 1.29 |
| 2008 sH1N1 dominance | 0.3452 | 0.3678 | -0.1452 | 0.7416 | 0.16 |
| 2009 H3N2 co-circulation | 1.0016 | 0.0001 | 1.6983 | 0.0000 | 10.50 |
| 2009 flu B co-circulation | 0.5510 | 0.0506 | 0.6060 | 0.0474 | 4.36 |
| Lower respiratory deaths | 0.0099 | 0.0000 | 0.0085 | 0.0001 | 0.82 |
| All registered take offs | -0.0001 | 0.4663 | -0.0002 | 0.3006 | 0.80 |

***Table B5 – All Age, Predictor Set 3,*** Matching

| **FACTOR NAMES** | **REGRESSION COEFFICIENT** | **P-VALUE UNDER H(0)** | **REGRESSION COEFFICIENT** | **P-VALUE UNDER H(1)** | **PROPORTION (%) BETWEEN COUNTRY VARIANCE REDUCTION** |
| --- | --- | --- | --- | --- | --- |
| Pm10 | 0.0062 | 0.0003 | 0.0065 | 0.0001 | 2.09 |
| Co2 emissions | 0.0000 | 0.8509 | 0.0000 | 0.8737 | 0.01 |
| Pop. density | 0.0002 | 0.0008 | 0.0002 | 0.0008 | 2.49 |
| Lattitude | -0.0160 | 0.0000 | -0.0163 | 0.0000 | 6.41 |
| % BMI >30 | -0.0061 | 0.1485 | -0.0057 | 0.1725 | 1.11 |
| % female BMI >30 | -0.0025 | 0.8899 | -0.0008 | 0.9621 | 0.00 |
| % male BMI >30 | -0.0694 | 0.0005 | -0.0648 | 0.0010 | 0.30 |
| Female COPD deaths | 0.0113 | 0.0000 | 0.0118 | 0.0000 | 0.36 |
| Male COPD deaths | 0.0061 | 0.0000 | 0.0063 | 0.0000 | 0.38 |
| % HIV | 0.0375 | 0.0024 | 0.0399 | 0.0008 | 1.43 |
| % TB | 0.0008 | 0.0000 | 0.0009 | 0.0000 | 0.02 |
| % HIV ARTs | 0.0130 | 0.3488 | 0.0133 | 0.3391 | 1.01 |
| % 13-14 yr olds with wheeze | -0.0135 | 0.5609 | -0.0136 | 0.5555 | 0.72 |
| Kilos of drugs distr. 2007-2009 | -0.0187 | 0.5525 | -0.0181 | 0.5660 | 0.56 |
| Kilos of drugs distr. 2009 | -0.0518 | 0.2515 | -0.0490 | 0.2813 | 1.95 |
| Health care spending as % GDP | -0.0625 | 0.0002 | -0.0628 | 0.0002 | 0.01 |
| % <15 | 0.0295 | 0.0000 | 0.0306 | 0.0000 | 28.57 |
| % > 60 | -0.0551 | 0.0000 | -0.0569 | 0.0000 | 42.70 |
| Crude birth rate | 0.0257 | 0.0000 | 0.0257 | 0.0000 | 0.00 |
| Pandemic Start | -0.0087 | 0.4021 | -0.0100 | 0.3119 | 1.32 |
| Pandemic Peak | -0.0132 | 0.1177 | -0.0139 | 0.0978 | 3.23 |
| 2008 H3N2 dominance | -0.5053 | 0.0017 | -0.5437 | 0.0001 | 4.45 |
| 2008 sH1N1 dominance | 0.2292 | 0.2706 | 0.2406 | 0.1891 | 2.64 |
| 2009 H3N2 co-circulation | 0.5211 | 0.0000 | 0.5524 | 0.0000 | 4.80 |
| 2009 flu B co-circulation | 0.4545 | 0.0009 | 0.4369 | 0.0002 | 3.91 |
| Lower respiratory deaths | 0.0026 | 0.0054 | 0.0027 | 0.0038 | 0.06 |
| All registered take offs | 0.0000 | 0.9812 | 0.0000 | 0.9514 | 0.00 |

***Table B6 – All Age, Predictor Set 3,*** Imputation

| **FACTOR NAMES** | **REGRESSION COEFFICIENT** | **P-VALUE UNDER H(0)** | **REGRESSION COEFFICIENT** | **P-VALUE UNDER H(1)** | **PROPORTION (%) BETWEEN COUNTRY VARIANCE** **REDUCTION** |
| --- | --- | --- | --- | --- | --- |
| Pm10 | 0.0080 | 0.0136 | 0.0163 | 0.0000 | 4.28 |
| Co2 emissions | -0.0003 | 0.0836 | -0.0001 | 0.2959 | 0.68 |
| Pop. density | 0.0001 | 0.3715 | 0.0001 | 0.3372 | 0.56 |
| Lattitude | -0.0330 | 0.0000 | -0.0274 | 0.0000 | 6.27 |
| % BMI >30 | -0.0282 | 0.0004 | -0.0227 | 0.0177 | 0.03 |
| % female BMI >30 | -0.0571 | 0.0938 | -0.0777 | 0.0421 | 0.11 |
| % male BMI >30 | -0.2060 | 0.0000 | -0.2589 | 0.0000 | 0.63 |
| Female COPD deaths | 0.0309 | 0.0000 | 0.0224 | 0.0000 | 0.10 |
| Male COPD deaths | 0.0161 | 0.0000 | 0.0195 | 0.0000 | 2.53 |
| % HIV | 0.1126 | 0.0000 | 0.0928 | 0.0000 | 3.05 |
| % TB | 0.0020 | 0.0000 | 0.0013 | 0.0001 | 0.04 |
| % HIV ARTs | 0.0714 | 0.0064 | 0.0356 | 0.1661 | 2.24 |
| % 13-14 yr olds with wheeze | -0.0184 | 0.5585 | -0.0334 | 0.2720 | 2.44 |
| Kilos of drugs distr. 2007-2009 | -0.0419 | 0.3538 | -0.0250 | 0.6087 | 0.42 |
| Kilos of drugs distr. 2009 | -0.1008 | 0.1175 | -0.0409 | 0.5631 | 0.58 |
| Health care spending as % GDP | -0.1050 | 0.0047 | -0.1410 | 0.0000 | 0.09 |
| % <15 | 0.0656 | 0.0000 | 0.0827 | 0.0000 | 48.65 |
| % > 60 | -0.1493 | 0.0000 | -0.1144 | 0.0000 | 35.80 |
| Crude birth rate | 0.0631 | 0.0000 | 0.0761 | 0.0000 | 6.87 |
| Pandemic Start | 0.0056 | 0.7501 | -0.0009 | 0.9581 | 0.01 |
| Pandemic Peak | -0.0202 | 0.1701 | -0.0075 | 0.6188 | 0.30 |
| 2008 H3N2 dominance | -0.8870 | 0.0011 | -1.2001 | 0.0000 | 10.14 |
| 2008 sH1N1 dominance | 0.5100 | 0.1451 | -0.1272 | 0.7624 | 0.13 |
| 2009 H3N2 co-circulation | 0.8000 | 0.0006 | 1.3129 | 0.0000 | 8.79 |
| 2009 flu B co-circulation | 0.4623 | 0.0699 | 0.1506 | 0.5581 | 0.42 |
| Lower respiratory deaths | 0.0062 | 0.0005 | 0.0087 | 0.0000 | 2.11 |
| All registered take offs | -0.0001 | 0.6186 | -0.0001 | 0.4189 | 0.51 |

***Table B7 – All Age, Predictor Set 4,*** Matching

| **FACTOR NAMES** | **REGRESSION COEFFICIENT** | **P-VALUE UNDER H(0)** | **REGRESSION COEFFICIENT** | **P-VALUE UNDER H(1)** | **PROPORTION (%) BETWEEN COUNTRY VARIANCE** **REDUCTION** |
| --- | --- | --- | --- | --- | --- |
| Pm10 | 0.0083 | 0.0000 | 0.0089 | 0.0000 | 5.16 |
| Co2 emissions | 0.0000 | 0.8053 | 0.0000 | 0.8581 | 0.01 |
| Pop. density | 0.0002 | 0.0055 | 0.0002 | 0.0061 | 0.29 |
| Lattitude | -0.0199 | 0.0000 | -0.0207 | 0.0000 | 12.56 |
| % BMI >30 | -0.0079 | 0.1025 | -0.0070 | 0.1419 | 1.28 |
| % female BMI >30 | -0.0001 | 0.9954 | 0.0036 | 0.8568 | 0.04 |
| % male BMI >30 | -0.0814 | 0.0004 | -0.0695 | 0.0016 | 1.30 |
| Female COPD deaths | 0.0126 | 0.0000 | 0.0137 | 0.0000 | -0.01 |
| Male COPD deaths | 0.0058 | 0.0001 | 0.0062 | 0.0000 | 0.69 |
| % HIV | 0.0305 | 0.0282 | 0.0374 | 0.0041 | 0.29 |
| % TB | 0.0009 | 0.0001 | 0.0010 | 0.0000 | 0.09 |
| % HIV ARTs | -0.0020 | 0.9014 | -0.0008 | 0.9598 | 0.00 |
| % 13-14 yr olds with wheeze | 0.0042 | 0.8635 | 0.0039 | 0.8712 | 0.07 |
| Kilos of drugs distr. 2007-2009 | -0.0282 | 0.4201 | -0.0265 | 0.4436 | 0.99 |
| Kilos of drugs distr. 2009 | -0.0695 | 0.1644 | -0.0627 | 0.2064 | 2.69 |
| Health care spending as % GDP | -0.0802 | 0.0000 | -0.0798 | 0.0000 | -0.02 |
| % <15 | 0.0361 | 0.0000 | 0.0385 | 0.0000 | 36.65 |
| % > 60 | -0.0652 | 0.0000 | -0.0698 | 0.0000 | 52.57 |
| Crude birth rate | 0.0309 | 0.0000 | 0.0305 | 0.0000 | 1.09 |
| Pandemic Start | -0.0137 | 0.2531 | -0.0160 | 0.1329 | 3.22 |
| Pandemic Peak | -0.0269 | 0.0056 | -0.0282 | 0.0030 | 4.09 |
| 2008 H3N2 dominance | -0.6282 | 0.0007 | -0.6977 | 0.0000 | 14.89 |
| 2008 sH1N1 dominance | 0.3121 | 0.1971 | 0.3303 | 0.0675 | 5.55 |
| 2009 H3N2 co-circulation | 0.6522 | 0.0000 | 0.6838 | 0.0000 | 18.01 |
| 2009 flu B co-circulation | 0.4641 | 0.0046 | 0.4132 | 0.0005 | 2.98 |
| Lower respiratory deaths | 0.0032 | 0.0033 | 0.0034 | 0.0015 | 0.04 |
| All registered take offs | 0.0000 | 0.8836 | 0.0000 | 0.8234 | 0.05 |

***Table B8 – All Age, Predictor Set 4,*** Imputation

| **FACTOR NAMES** | **REGRESSION COEFFICIENT** | **P-VALUE UNDER H(0)** | **REGRESSION COEFFICIENT** | **P-VALUE UNDER H(1)** | **PROPORTION (%) BETWEEN COUNTRY VARIANCE** **REDUCTION** |
| --- | --- | --- | --- | --- | --- |
| Pm10 | 0.0092 | 0.0027 | 0.0050 | 0.1022 | 1.72 |
| Co2 emissions | -0.0001 | 0.3281 | -0.0001 | 0.5873 | 0.18 |
| Pop. density | 0.0002 | 0.1527 | 0.0002 | 0.0479 | 2.27 |
| Lattitude | -0.0304 | 0.0000 | -0.0311 | 0.0000 | 8.20 |
| % BMI >30 | -0.0196 | 0.0074 | -0.0093 | 0.2349 | 0.85 |
| % female BMI >30 | -0.0159 | 0.6117 | 0.0087 | 0.7873 | 0.04 |
| % male BMI >30 | -0.1375 | 0.0001 | -0.0845 | 0.0172 | 0.07 |
| Female COPD deaths | 0.0155 | 0.0000 | 0.0186 | 0.0000 | 1.24 |
| Male COPD deaths | 0.0083 | 0.0003 | 0.0116 | 0.0000 | 0.76 |
| % HIV | 0.0523 | 0.0102 | 0.0652 | 0.0031 | 0.91 |
| % TB | 0.0014 | 0.0001 | 0.0017 | 0.0000 | 2.62 |
| % HIV ARTs | 0.0137 | 0.5756 | 0.0168 | 0.5258 | 0.45 |
| % 13-14 yr olds with wheeze | -0.0145 | 0.6492 | -0.0072 | 0.8315 | 0.09 |
| Kilos of drugs distr. 2007-2009 | -0.0392 | 0.3870 | -0.0263 | 0.5839 | 0.51 |
| Kilos of drugs distr. 2009 | -0.0971 | 0.1337 | -0.0737 | 0.2696 | 2.04 |
| Health care spending as % GDP | -0.0963 | 0.0047 | -0.1026 | 0.0013 | 0.04 |
| % <15 | 0.0475 | 0.0000 | 0.0598 | 0.0000 | 26.30 |
| % > 60 | -0.0886 | 0.0000 | -0.0591 | 0.0000 | 14.07 |
| Crude birth rate | 0.0372 | 0.0000 | 0.0380 | 0.0000 | 0.02 |
| Pandemic Start | -0.0086 | 0.6243 | -0.0284 | 0.1054 | 3.25 |
| Pandemic Peak | -0.0367 | 0.0101 | -0.0460 | 0.0045 | 4.14 |
| 2008 H3N2 dominance | -0.8474 | 0.0013 | -0.9014 | 0.0006 | 6.61 |
| 2008 sH1N1 dominance | 0.2710 | 0.4283 | -0.0827 | 0.8383 | 0.06 |
| 2009 H3N2 co-circulation | 0.9246 | 0.0000 | 1.2536 | 0.0000 | 11.60 |
| 2009 flu B co-circulation | 0.7430 | 0.0014 | 0.7561 | 0.0026 | 1.76 |
| Lower respiratory deaths | 0.0046 | 0.0057 | 0.0084 | 0.0000 | 2.30 |
| All registered take offs | -0.0001 | 0.5765 | -0.0001 | 0.4996 | 0.34 |

***Table B9 – <65, Predictor Set 1,*** Matching

| **FACTOR NAMES** | **REGRESSION COEFFICIENT** | **P-VALUE UNDER H(0)** | **REGRESSION COEFFICIENT** | **P-VALUE UNDER H(1)** | **PROPORTION (%) BETWEEN COUNTRY VARIANCE** **REDUCTION** |
| --- | --- | --- | --- | --- | --- |
| Pm10 | 0.0056 | 0.0004 | 0.0054 | 0.0006 | 1.22 |
| Co2 emissions | -0.0001 | 0.4031 | 0.0000 | 0.4869 | 0.32 |
| Pop. density | -0.0001 | 0.1302 | -0.0001 | 0.1052 | 1.70 |
| Lattitude | -0.0163 | 0.0000 | -0.0151 | 0.0000 | 11.99 |
| % BMI >30 | -0.0118 | 0.0037 | -0.0074 | 0.0619 | 1.58 |
| % female BMI >30 | -0.0214 | 0.2164 | -0.0065 | 0.6924 | -0.10 |
| % male BMI >30 | -0.0880 | 0.0000 | -0.0776 | 0.0000 | -0.11 |
| Female COPD deaths | 0.0102 | 0.0000 | 0.0110 | 0.0000 | 0.01 |
| Male COPD deaths | 0.0067 | 0.0000 | 0.0067 | 0.0000 | 1.84 |
| % HIV | 0.0432 | 0.0000 | 0.0542 | 0.0000 | 11.37 |
| % TB | 0.0008 | 0.0000 | 0.0010 | 0.0000 | 2.02 |
| % HIV ARTs | 0.0302 | 0.0331 | 0.0305 | 0.0323 | 3.63 |
| % 13-14 yr olds with wheeze | 0.0112 | 0.4094 | 0.0109 | 0.4211 | 1.27 |
| Kilos of drugs distr. 2007-2009 | -0.0252 | 0.3250 | -0.0253 | 0.3186 | 1.81 |
| Kilos of drugs distr. 2009 | -0.0506 | 0.1671 | -0.0534 | 0.1506 | 3.60 |
| Health care spending as % GDP | -0.0329 | 0.0385 | -0.0337 | 0.0329 | 4.76 |
| % <15 | 0.0277 | 0.0000 | 0.0302 | 0.0000 | 34.10 |
| % > 60 | -0.0494 | 0.0000 | -0.0538 | 0.0000 | 49.21 |
| Crude birth rate | 0.0320 | 0.0000 | 0.0337 | 0.0000 | 3.01 |
| Pandemic Start | -0.0118 | 0.1526 | -0.0116 | 0.1224 | 3.29 |
| Pandemic Peak | -0.0187 | 0.0061 | -0.0195 | 0.0036 | 2.93 |
| 2008 H3N2 dominance | -0.3609 | 0.0072 | -0.3645 | 0.0002 | 5.67 |
| 2008 sH1N1 dominance | 0.1067 | 0.5345 | 0.1088 | 0.3701 | 1.29 |
| 2009 H3N2 co-circulation | 0.3785 | 0.0006 | 0.3506 | 0.0000 | 5.10 |
| 2009 flu B co-circulation | 0.2886 | 0.0147 | 0.2654 | 0.0009 | 3.25 |
| Lower respiratory deaths | 0.0033 | 0.0002 | 0.0034 | 0.0001 | 1.91 |
| All registered take offs | 0.0000 | 0.6267 | 0.0000 | 0.5231 | 0.38 |

***Table B10 – <65, Predictor Set 1,*** Imputation

| **FACTOR NAMES** | **REGRESSION COEFFICIENT** | **P-VALUE UNDER H(0)** | **REGRESSION COEFFICIENT** | **P-VALUE UNDER H(1)** | **PROPORTION (%) BETWEEN COUNTRY VARIANCE** **REDUCTION** |
| --- | --- | --- | --- | --- | --- |
| Pm10 | 0.0079 | 0.0003 | 0.0063 | 0.0127 | 0.00 |
| Co2 emissions | -0.0001 | 0.0846 | -0.0002 | 0.0352 | 0.35 |
| Pop. density | -0.0002 | 0.0405 | -0.0002 | 0.0460 | 1.06 |
| Lattitude | -0.0187 | 0.0000 | -0.0216 | 0.0000 | 7.09 |
| % BMI >30 | -0.0117 | 0.0468 | -0.0044 | 0.4399 | 0.36 |
| % female BMI >30 | -0.0044 | 0.8603 | 0.0344 | 0.1194 | 1.45 |
| % male BMI >30 | -0.1060 | 0.0001 | -0.0839 | 0.0015 | 0.41 |
| Female COPD deaths | 0.0106 | 0.0000 | 0.0162 | 0.0000 | 1.96 |
| Male COPD deaths | 0.0056 | 0.0008 | 0.0089 | 0.0000 | 0.00 |
| % HIV | 0.0439 | 0.0055 | 0.0435 | 0.0026 | 0.65 |
| % TB | 0.0010 | 0.0000 | 0.0011 | 0.0000 | 2.14 |
| % HIV ARTs | 0.0145 | 0.4588 | 0.0195 | 0.2629 | 1.38 |
| % 13-14 yr olds with wheeze | 0.0099 | 0.6107 | 0.0117 | 0.5428 | 0.75 |
| Kilos of drugs distr. 2007-2009 | -0.0579 | 0.0955 | -0.0648 | 0.0676 | 5.40 |
| Kilos of drugs distr. 2009 | -0.0891 | 0.0751 | -0.0834 | 0.0829 | 4.89 |
| Health care spending as % GDP | -0.0419 | 0.0586 | -0.0543 | 0.0097 | 3.95 |
| % <15 | 0.0462 | 0.0000 | 0.0437 | 0.0000 | 28.71 |
| % > 60 | -0.0824 | 0.0000 | -0.0682 | 0.0000 | 36.33 |
| Crude birth rate | 0.0342 | 0.0000 | 0.0372 | 0.0000 | 0.22 |
| Pandemic Start | -0.0090 | 0.4740 | -0.0110 | 0.3775 | 0.99 |
| Pandemic Peak | -0.0169 | 0.1060 | -0.0173 | 0.1301 | 2.68 |
| 2008 H3N2 dominance | -0.5218 | 0.0115 | -0.6814 | 0.0010 | 3.35 |
| 2008 sH1N1 dominance | 0.1151 | 0.6611 | -0.3704 | 0.2050 | 2.34 |
| 2009 H3N2 co-circulation | 0.4611 | 0.0066 | 0.7378 | 0.0000 | 2.54 |
| 2009 flu B co-circulation | 0.2883 | 0.1121 | 0.1662 | 0.4206 | 0.74 |
| Lower respiratory deaths | 0.0033 | 0.0063 | 0.0050 | 0.0001 | 1.55 |
| All registered take offs | 0.0000 | 0.5637 | -0.0001 | 0.3304 | 0.71 |

***Table B11 – <65, Predictor Set 2,*** Matching

| **FACTOR NAMES** | **REGRESSION COEFFICIENT** | **P-VALUE UNDER H(0)** | **REGRESSION COEFFICIENT** | **P-VALUE UNDER H(1)** | **PROPORTION (%) BETWEEN COUNTRY VARIANCE** **REDUCTION** |
| --- | --- | --- | --- | --- | --- |
| Pm10 | 0.0055 | 0.0006 | 0.0054 | 0.0007 | 0.75 |
| Co2 emissions | -0.0001 | 0.2606 | -0.0001 | 0.2919 | 0.69 |
| Pop. density | -0.0001 | 0.0361 | -0.0001 | 0.0296 | 1.30 |
| Lattitude | -0.0157 | 0.0000 | -0.0151 | 0.0000 | 8.18 |
| % BMI >30 | -0.0089 | 0.0253 | -0.0068 | 0.0838 | 1.69 |
| % female BMI >30 | -0.0155 | 0.3606 | -0.0086 | 0.6031 | 0.12 |
| % male BMI >30 | -0.0872 | 0.0000 | -0.0821 | 0.0000 | 0.02 |
| Female COPD deaths | 0.0118 | 0.0000 | 0.0122 | 0.0000 | 0.40 |
| Male COPD deaths | 0.0076 | 0.0000 | 0.0076 | 0.0000 | 3.35 |
| % HIV | 0.0540 | 0.0000 | 0.0579 | 0.0000 | 11.31 |
| % TB | 0.0010 | 0.0000 | 0.0011 | 0.0000 | 1.69 |
| % HIV ARTs | 0.0368 | 0.0047 | 0.0367 | 0.0048 | 6.10 |
| % 13-14 yr olds with wheeze | -0.0034 | 0.8040 | -0.0033 | 0.8084 | 0.14 |
| Kilos of drugs distr. 2007-2009 | -0.0366 | 0.1672 | -0.0365 | 0.1666 | 3.26 |
| Kilos of drugs distr. 2009 | -0.0661 | 0.0811 | -0.0675 | 0.0772 | 5.17 |
| Health care spending as % GDP | -0.0492 | 0.0019 | -0.0493 | 0.0017 | 1.58 |
| % <15 | 0.0309 | 0.0000 | 0.0322 | 0.0000 | 35.68 |
| % > 60 | -0.0560 | 0.0000 | -0.0583 | 0.0000 | 51.29 |
| Crude birth rate | 0.0326 | 0.0000 | 0.0326 | 0.0000 | 1.42 |
| Pandemic Start | -0.0057 | 0.5218 | -0.0060 | 0.4757 | 0.68 |
| Pandemic Peak | -0.0109 | 0.1414 | -0.0115 | 0.1170 | 2.94 |
| 2008 H3N2 dominance | -0.4072 | 0.0038 | -0.4098 | 0.0005 | 2.14 |
| 2008 sH1N1 dominance | 0.1401 | 0.4390 | 0.1434 | 0.3372 | 1.44 |
| 2009 H3N2 co-circulation | 0.4176 | 0.0003 | 0.3975 | 0.0000 | 0.67 |
| 2009 flu B co-circulation | 0.4063 | 0.0008 | 0.3853 | 0.0001 | 5.30 |
| Lower respiratory deaths | 0.0032 | 0.0003 | 0.0033 | 0.0002 | 0.66 |
| All registered take offs | 0.0000 | 0.8606 | 0.0000 | 0.9293 | -0.01 |

***Table B12 – <65, Predictor Set 2,*** Imputation

| **FACTOR NAMES** | **REGRESSION COEFFICIENT** | **P-VALUE UNDER H(0)** | **REGRESSION COEFFICIENT** | **P-VALUE UNDER H(1)** | **PROPORTION (%) BETWEEN COUNTRY VARIANCE** **REDUCTION** |
| --- | --- | --- | --- | --- | --- |
| Pm10 | 0.0137 | 0.0000 | 0.0163 | 0.0000 | 4.99 |
| Co2 emissions | -0.0003 | 0.0138 | -0.0003 | 0.0136 | 0.45 |
| Pop. density | -0.0003 | 0.0085 | -0.0003 | 0.0096 | 1.56 |
| Lattitude | -0.0257 | 0.0000 | -0.0306 | 0.0000 | 4.65 |
| % BMI >30 | -0.0307 | 0.0000 | -0.0293 | 0.0003 | 0.36 |
| % female BMI >30 | -0.0783 | 0.0109 | -0.0917 | 0.0115 | 0.81 |
| % male BMI >30 | -0.2328 | 0.0000 | -0.2313 | 0.0000 | 2.36 |
| Female COPD deaths | 0.0267 | 0.0000 | 0.0247 | 0.0000 | 0.01 |
| Male COPD deaths | 0.0150 | 0.0000 | 0.0166 | 0.0000 | 0.89 |
| % HIV | 0.0972 | 0.0000 | 0.0850 | 0.0002 | 0.71 |
| % TB | 0.0026 | 0.0000 | 0.0028 | 0.0000 | 6.37 |
| % HIV ARTs | 0.0567 | 0.0115 | 0.0570 | 0.0128 | 2.93 |
| % 13-14 yr olds with wheeze | -0.0155 | 0.5327 | -0.0238 | 0.3337 | 1.89 |
| Kilos of drugs distr. 2007-2009 | -0.0891 | 0.0227 | -0.0786 | 0.0330 | 0.12 |
| Kilos of drugs distr. 2009 | -0.1431 | 0.0105 | -0.1375 | 0.0187 | 0.71 |
| Health care spending as % GDP | -0.1726 | 0.0000 | -0.1636 | 0.0000 | 0.06 |
| % <15 | 0.0636 | 0.0000 | 0.0845 | 0.0000 | 55.83 |
| % > 60 | -0.1266 | 0.0000 | -0.1414 | 0.0000 | 64.01 |
| Crude birth rate | 0.0849 | 0.0000 | 0.0866 | 0.0000 | 10.65 |
| Pandemic Start | 0.0190 | 0.2722 | 0.0019 | 0.8956 | 0.02 |
| Pandemic Peak | -0.0096 | 0.5147 | -0.0210 | 0.1532 | 2.36 |
| 2008 H3N2 dominance | -0.7774 | 0.0048 | -0.7735 | 0.0028 | 0.01 |
| 2008 sH1N1 dominance | 0.3654 | 0.2995 | -0.0529 | 0.8939 | 0.02 |
| 2009 H3N2 co-circulation | 0.8749 | 0.0002 | 1.1795 | 0.0000 | 4.13 |
| 2009 flu B co-circulation | 0.7476 | 0.0029 | 0.6611 | 0.0062 | 0.00 |
| Lower respiratory deaths | 0.0095 | 0.0000 | 0.0095 | 0.0000 | 4.44 |
| All registered take offs | -0.0002 | 0.1288 | -0.0002 | 0.1146 | 1.83 |

***Table B13 – <65, Predictor Set 3, Matching***

| **FACTOR NAMES** | **REGRESSION COEFFICIENT** | **P-VALUE UNDER H(0)** | **REGRESSION COEFFICIENT** | **P-VALUE UNDER H(1)** | **PROPORTION (%) BETWEEN COUNTRY VARIANCE** **REDUCTION** |
| --- | --- | --- | --- | --- | --- |
| Pm10 | 0.0052 | 0.0004 | 0.0051 | 0.0005 | 1.28 |
| Co2 emissions | -0.0001 | 0.3351 | -0.0001 | 0.3661 | 0.50 |
| Pop. density | -0.0001 | 0.0491 | -0.0001 | 0.0451 | 1.23 |
| Lattitude | -0.0123 | 0.0000 | -0.0120 | 0.0000 | 4.40 |
| % BMI >30 | -0.0045 | 0.2190 | -0.0030 | 0.4063 | 0.38 |
| % female BMI >30 | -0.0035 | 0.8221 | 0.0012 | 0.9354 | 0.01 |
| % male BMI >30 | -0.0610 | 0.0004 | -0.0583 | 0.0006 | 0.31 |
| Female COPD deaths | 0.0096 | 0.0000 | 0.0099 | 0.0000 | 0.16 |
| Male COPD deaths | 0.0056 | 0.0000 | 0.0056 | 0.0000 | 0.67 |
| % HIV | 0.0397 | 0.0001 | 0.0429 | 0.0000 | 5.22 |
| % TB | 0.0008 | 0.0000 | 0.0008 | 0.0000 | 0.59 |
| % HIV ARTs | 0.0234 | 0.0669 | 0.0235 | 0.0647 | 3.71 |
| % 13-14 yr olds with wheeze | -0.0028 | 0.8398 | -0.0028 | 0.8412 | 0.08 |
| Kilos of drugs distr. 2007-2009 | -0.0359 | 0.1833 | -0.0359 | 0.1829 | 2.97 |
| Kilos of drugs distr. 2009 | -0.0662 | 0.0865 | -0.0673 | 0.0833 | 4.90 |
| Health care spending as % GDP | -0.0448 | 0.0019 | -0.0450 | 0.0018 | 0.93 |
| % <15 | 0.0251 | 0.0000 | 0.0261 | 0.0000 | 27.85 |
| % > 60 | -0.0486 | 0.0000 | -0.0504 | 0.0000 | 45.15 |
| Crude birth rate | 0.0248 | 0.0000 | 0.0256 | 0.0000 | 0.06 |
| Pandemic Start | -0.0052 | 0.5362 | -0.0054 | 0.5053 | 0.57 |
| Pandemic Peak | -0.0086 | 0.2129 | -0.0090 | 0.1878 | 2.08 |
| 2008 H3N2 dominance | -0.3713 | 0.0058 | -0.3779 | 0.0016 | 1.25 |
| 2008 sH1N1 dominance | 0.1575 | 0.3595 | 0.1583 | 0.2936 | 1.70 |
| 2009 H3N2 co-circulation | 0.3577 | 0.0008 | 0.3538 | 0.0002 | 0.17 |
| 2009 flu B co-circulation | 0.3212 | 0.0046 | 0.3129 | 0.0013 | 1.62 |
| Lower respiratory deaths | 0.0023 | 0.0055 | 0.0023 | 0.0041 | 0.06 |
| All registered take offs | 0.0000 | 0.9525 | 0.0000 | 0.9956 | -0.01 |

***Table B14 – <65, Predictor Set 3,*** Imputation

| **FACTOR NAMES** | **REGRESSION COEFFICIENT** | **P-VALUE UNDER H(0)** | **REGRESSION COEFFICIENT** | **P-VALUE UNDER H(1)** | **PROPORTION (%) BETWEEN COUNTRY VARIANCE** **REDUCTION** |
| --- | --- | --- | --- | --- | --- |
| Pm10 | 0.0101 | 0.0005 | 0.0102 | 0.0013 | 0.47 |
| Co2 emissions | -0.0003 | 0.0240 | -0.0003 | 0.0195 | 0.50 |
| Pop. density | -0.0003 | 0.0078 | -0.0003 | 0.0147 | 1.78 |
| Lattitude | -0.0207 | 0.0000 | -0.0223 | 0.0000 | 2.49 |
| % BMI >30 | -0.0335 | 0.0000 | -0.0272 | 0.0003 | 0.87 |
| % female BMI >30 | -0.0828 | 0.0163 | -0.0439 | 0.1431 | 1.29 |
| % male BMI >30 | -0.2503 | 0.0000 | -0.2441 | 0.0000 | 2.89 |
| Female COPD deaths | 0.0225 | 0.0000 | 0.0292 | 0.0000 | 0.97 |
| Male COPD deaths | 0.0156 | 0.0000 | 0.0191 | 0.0000 | 4.81 |
| % HIV | 0.1051 | 0.0000 | 0.1097 | 0.0000 | 7.31 |
| % TB | 0.0028 | 0.0000 | 0.0028 | 0.0000 | 7.55 |
| % HIV ARTs | 0.0720 | 0.0013 | 0.0712 | 0.0003 | 10.29 |
| % 13-14 yr olds with wheeze | -0.0198 | 0.3874 | -0.0287 | 0.2348 | 2.79 |
| Kilos of drugs distr. 2007-2009 | -0.0713 | 0.0504 | -0.0867 | 0.0315 | 7.28 |
| Kilos of drugs distr. 2009 | -0.1164 | 0.0257 | -0.1198 | 0.0335 | 0.94 |
| Health care spending as % GDP | -0.1489 | 0.0000 | -0.1505 | 0.0000 | 0.00 |
| % <15 | 0.0657 | 0.0000 | 0.0668 | 0.0000 | 46.56 |
| % > 60 | -0.1091 | 0.0000 | -0.1532 | 0.0000 | 63.32 |
| Crude birth rate | 0.0719 | 0.0000 | 0.0908 | 0.0000 | 10.62 |
| Pandemic Start | 0.0184 | 0.2326 | 0.0043 | 0.7713 | 0.11 |
| Pandemic Peak | -0.0060 | 0.6430 | -0.0041 | 0.7771 | 0.09 |
| 2008 H3N2 dominance | -0.7828 | 0.0014 | -1.0202 | 0.0001 | 4.97 |
| 2008 sH1N1 dominance | 0.4726 | 0.1336 | 0.2566 | 0.5095 | 0.64 |
| 2009 H3N2 co-circulation | 0.7649 | 0.0002 | 0.9890 | 0.0000 | 3.41 |
| 2009 flu B co-circulation | 0.6472 | 0.0034 | 0.6276 | 0.0104 | 0.04 |
| Lower respiratory deaths | 0.0068 | 0.0000 | 0.0081 | 0.0000 | 4.34 |
| All registered take offs | -0.0002 | 0.1007 | -0.0002 | 0.1260 | 1.73 |

***Table B15 – <65, Predictor Set 4, Matching***

| **FACTOR NAMES** | **REGRESSION COEFFICIENT** | **P-VALUE UNDER H(0)** | **REGRESSION COEFFICIENT** | **P-VALUE UNDER H(1)** | **PROPORTION (%) BETWEEN COUNTRY VARIANCE** **REDUCTION** |
| --- | --- | --- | --- | --- | --- |
| Pm10 | 0.0051 | 0.0004 | 0.0049 | 0.0006 | 2.52 |
| Co2 emissions | 0.0000 | 0.5670 | 0.0000 | 0.6675 | 0.10 |
| Pop. density | -0.0001 | 0.2624 | -0.0001 | 0.2223 | 0.99 |
| Lattitude | -0.0105 | 0.0000 | -0.0099 | 0.0000 | 4.26 |
| % BMI >30 | 0.0021 | 0.5550 | 0.0049 | 0.1592 | 1.41 |
| % female BMI >30 | 0.0207 | 0.1598 | 0.0297 | 0.0385 | 3.09 |
| % male BMI >30 | -0.0227 | 0.1858 | -0.0203 | 0.2216 | 0.94 |
| Female COPD deaths | 0.0061 | 0.0003 | 0.0069 | 0.0000 | 0.27 |
| Male COPD deaths | 0.0021 | 0.0560 | 0.0023 | 0.0364 | 3.12 |
| % HIV | 0.0193 | 0.0545 | 0.0275 | 0.0048 | 6.00 |
| % TB | 0.0004 | 0.0126 | 0.0005 | 0.0010 | 0.07 |
| % HIV ARTs | 0.0027 | 0.8509 | 0.0034 | 0.8089 | 0.10 |
| % 13-14 yr olds with wheeze | 0.0058 | 0.6849 | 0.0058 | 0.6848 | 0.37 |
| Kilos of drugs distr. 2007-2009 | -0.0372 | 0.1721 | -0.0370 | 0.1713 | 3.19 |
| Kilos of drugs distr. 2009 | -0.0690 | 0.0765 | -0.0708 | 0.0713 | 5.42 |
| Health care spending as % GDP | -0.0397 | 0.0049 | -0.0402 | 0.0044 | 0.42 |
| % <15 | 0.0167 | 0.0000 | 0.0191 | 0.0000 | 17.61 |
| % > 60 | -0.0378 | 0.0000 | -0.0423 | 0.0000 | 37.65 |
| Crude birth rate | 0.0166 | 0.0000 | 0.0185 | 0.0000 | 2.04 |
| Pandemic Start | -0.0109 | 0.1815 | -0.0107 | 0.1625 | 2.64 |
| Pandemic Peak | -0.0135 | 0.0470 | -0.0143 | 0.0324 | 0.72 |
| 2008 H3N2 dominance | -0.3178 | 0.0206 | -0.3465 | 0.0016 | 2.33 |
| 2008 sH1N1 dominance | 0.1506 | 0.3847 | 0.1495 | 0.2690 | 2.10 |
| 2009 H3N2 co-circulation | 0.3288 | 0.0020 | 0.3346 | 0.0000 | 2.11 |
| 2009 flu B co-circulation | 0.2229 | 0.0509 | 0.2169 | 0.0111 | 8.02 |
| Lower respiratory deaths | 0.0011 | 0.1792 | 0.0013 | 0.1158 | 1.64 |
| All registered take offs | 0.0000 | 0.7086 | 0.0000 | 0.6231 | 0.23 |

***Table B16 – <65, Predictor Set 4,*** Imputation

| **FACTOR NAMES** | **REGRESSION COEFFICIENT** | **P-VALUE UNDER H(0)** | **REGRESSION COEFFICIENT** | **P-VALUE UNDER H(1)** | **PROPORTION (%) BETWEEN COUNTRY VARIANCE** **REDUCTION** |
| --- | --- | --- | --- | --- | --- |
| Pm10 | 0.0075 | 0.0046 | 0.0072 | 0.0067 | 0.21 |
| Co2 emissions | -0.0002 | 0.0218 | -0.0002 | 0.0613 | 2.10 |
| Pop. density | -0.0002 | 0.0267 | -0.0002 | 0.0498 | 1.33 |
| Lattitude | -0.0305 | 0.0000 | -0.0207 | 0.0000 | 17.79 |
| % BMI >30 | -0.0146 | 0.0220 | -0.0104 | 0.0906 | 1.67 |
| % female BMI >30 | -0.0044 | 0.8722 | -0.0098 | 0.7256 | 0.07 |
| % male BMI >30 | -0.1215 | 0.0001 | -0.1038 | 0.0005 | 0.75 |
| Female COPD deaths | 0.0153 | 0.0000 | 0.0230 | 0.0000 | 2.06 |
| Male COPD deaths | 0.0078 | 0.0001 | 0.0106 | 0.0000 | 0.01 |
| % HIV | 0.0647 | 0.0002 | 0.0805 | 0.0000 | 7.24 |
| % TB | 0.0013 | 0.0000 | 0.0022 | 0.0000 | 4.59 |
| % HIV ARTs | 0.0369 | 0.0749 | 0.0452 | 0.0229 | 3.06 |
| % 13-14 yr olds with wheeze | 0.0036 | 0.8709 | -0.0009 | 0.9663 | 0.00 |
| Kilos of drugs distr. 2007-2009 | -0.0711 | 0.0635 | -0.0643 | 0.0794 | 4.98 |
| Kilos of drugs distr. 2009 | -0.1188 | 0.0304 | -0.1356 | 0.0248 | 0.94 |
| Health care spending as % GDP | -0.0786 | 0.0049 | -0.0672 | 0.0090 | 1.71 |
| % <15 | 0.0468 | 0.0000 | 0.0543 | 0.0000 | 33.07 |
| % > 60 | -0.0908 | 0.0000 | -0.0845 | 0.0000 | 38.16 |
| Crude birth rate | 0.0432 | 0.0000 | 0.0716 | 0.0000 | 0.16 |
| Pandemic Start | 0.0020 | 0.8972 | -0.0050 | 0.7197 | 0.16 |
| Pandemic Peak | -0.0278 | 0.0284 | -0.0239 | 0.0651 | 3.92 |
| 2008 H3N2 dominance | -0.6661 | 0.0058 | -0.7217 | 0.0056 | 0.01 |
| 2008 sH1N1 dominance | 0.3033 | 0.3241 | -0.0042 | 0.9899 | 0.00 |
| 2009 H3N2 co-circulation | 0.7731 | 0.0000 | 1.1366 | 0.0000 | 13.90 |
| 2009 flu B co-circulation | 0.7296 | 0.0003 | 0.6492 | 0.0009 | 1.91 |
| Lower respiratory deaths | 0.0041 | 0.0040 | 0.0059 | 0.0001 | 1.28 |
| All registered take offs | -0.0001 | 0.5350 | -0.0001 | 0.3153 | 0.77 |

***Table C(1-2)* Regressions based on stage 1 sample (20 countries only)**

In the main text of the article we explained why doing traditional regression procedures (using all country pandemic mortality excess rates (170 rates)) fails. An alternative suggestion might be to only use the 20 countries with the observed excess mortality rates and doing 27 regressions (one for each factor, see Table C1) with the predictors (factors). Since the variation is dominated by the age effect, we also ran a second regression with age as a confounder variable. The advantage is that if a predictor caused the variation, its effect should be present in these 20 observed rates.

The main problem with this approach is that there are only 20 country values and the sample is not a random sample of world countries. In such a situation, many things can go wrong and spurious findings will be found due to:

- Outlier values on the predictor dataset (e.g. antivirals causing higher mortality (see all age, corrected model)
- The non-random nature of the sampled countries (the effect of the pandemic start is due to over sampling the first hit countries (Mexico and USA) and the southern hemisphere Latin American countries) with the winter in July-August (Argentina).
- Missing effects due to the small and non-representativeness of the sample for the predictor variable (for instance the relation with other immune compromising effects like TB, HIV, or the interactions with the types of influenza virus).

For completeness we provided the results below (see tables C).

**Tables C (1-2) – Stage I countries-only results**

***Table C1 – All Age***

| **TABLE C-ALL AGES** | | **RESULTS WITH THE FACTOR -ONLY** | | **RESULTS WITH THE FACTOR AND ADJUSTMENT FOR AGE** | |
| --- | --- | --- | --- | --- | --- |
| FACTOR NAMES | EXPECTED DIRECTION OF EFFECT | REGRESSION COEFFICIENT | P-VALUE | REGRESSION COEFFICIENT | P-VALUE |
| Pm10 | **+** | 0.0514 | 0.0136 | 0.0372 | 0.1013 |
| Co2 emissions | **+** | 0.0001 | 0.7532 | 0.0002 | 0.3522 |
| Pop. density | **+** | 0.0004 | 0.0884 | -0.0001 | 0.5088 |
| Lattitude | **+** | -0.0481 | 0.0732 | -0.0183 | 0.3476 |
| % BMI >30 | **+** | 0.0517 | 0.1559 | 0.0218 | 0.5593 |
| % female BMI >30 | **+** | 0.2326 | 0.1142 | 0.0757 | 0.6362 |
| % male BMI >30 | **+** | 0.2342 | 0.2220 | 0.1100 | 0.5362 |
| Female COPD deaths | **+** | 0.0474 | 0.0129 | 0.0268 | 0.3444 |
| Male COPD deaths | **+** | 0.0166 | 0.2276 | -0.0077 | 0.6387 |
| % HIV | **+** | 0.0891 | 0.3112 | 0.0134 | 0.8879 |
| % TB | **+** | 0.0019 | 0.3268 | 0.0002 | 0.9225 |
| % HIV ARTs | **-** | -0.0615 | 0.8058 | -0.2495 | 0.1646 |
| % 13-14 yr olds with wheeze | **+** | -0.0525 | 0.4945 | -0.0147 | 0.7294 |
| Kilos of drugs distr. 2007-2009 | **-** | 0.0534 | 0.5508 | 0.1659 | 0.0243 |
| Kilos of drugs distr. 2009 | **-** | 0.0568 | 0.7731 | 0.3494 | 0.0396 |
| Health care spending as % GDP | **-** | -0.0070 | 0.9572 | 0.0730 | 0.5289 |
| % <15 | **+** | 0.1006 | 0.1250 | 0.0218 | 0.7608 |
| % > 60 | **-** | -0.1171 | 0.0097 | -0.1079 | 0.0463 |
| Crude birth rate | **+** | 0.1503 | 0.1886 | 0.0574 | 0.7082 |
| Pandemic Start | **+** | -0.1263 | 0.0017 | -0.1008 | 0.0066 |
| Pandemic Peak | **+** | 0.0007 | 0.9851 | 0.0391 | 0.2700 |
| 2008 H3N2 dominance | **-** | 0.1731 | 0.8047 | 0.5230 | 0.4072 |
| 2008 sH1N1 dominance | **+** | 0.1731 | 0.8047 | 0.5230 | 0.4072 |
| 2009 H3N2 co-circulation | **+** | -0.1523 | 0.8408 | -0.3466 | 0.6169 |
| 2009 flu B co-circulation | **+** | 0.1489 | 0.8377 | -0.0525 | 0.9326 |
| Lower respiratory deaths | **+** | 0.0055 | 0.6599 | 0.0118 | 0.2841 |
| All registered take offs | **+** | 0.0001 | 0.8136 | 0.0001 | 0.3653 |

Table C2 - <65

| **TABLE C-<65 AGES** | | **RESULTS WITH THE FACTOR -ONLY** | | **RESULTS WITH THE FACTOR AND ADJUSTMENT FOR AGE** | |
| --- | --- | --- | --- | --- | --- |
| FACTOR NAMES | EXPECTED DIRECTION OF EFFECT | REGRESSION COEFFICIENT | P-VALUE | REGRESSION COEFFICIENT | P-VALUE |
| Pm10 | **+** | 0.0333 | 0.0853 | 0.0067 | 0.7257 |
| Co2 emissions | **+** | 0.0000 | 0.9489 | 0.0000 | 0.9597 |
| Pop. density | **+** | -0.0001 | 0.6787 | -0.0001 | 0.6955 |
| Lattitude | **+** | -0.0209 | 0.2514 | -0.0118 | 0.4670 |
| % BMI >30 | **+** | 0.0715 | 0.0139 | 0.0498 | 0.0682 |
| % female BMI >30 | **+** | 0.2918 | 0.0139 | 0.1616 | 0.1814 |
| % male BMI >30 | **+** | 0.2774 | 0.0851 | 0.1438 | 0.2948 |
| Female COPD deaths | **+** | 0.0406 | 0.0147 | 0.0020 | 0.9311 |
| Male COPD deaths | **+** | 0.0134 | 0.2651 | -0.0169 | 0.1706 |
| % HIV | **+** | 0.0999 | 0.1838 | 0.0425 | 0.5645 |
| % TB | **+** | 0.0018 | 0.2642 | 0.0003 | 0.8268 |
| % HIV ARTs | **-** | -0.0190 | 0.9334 | -0.0986 | 0.6015 |
| % 13-14 yr olds with wheeze | **+** | -0.0015 | 0.9708 | 0.0065 | 0.8291 |
| Kilos of drugs distr. 2007-2009 | **-** | -0.0333 | 0.6699 | 0.0679 | 0.2851 |
| Kilos of drugs distr. 2009 | **-** | -0.1414 | 0.3986 | 0.1080 | 0.4598 |
| Health care spending as % GDP | **-** | 0.0182 | 0.8732 | 0.1116 | 0.2064 |
| % <15 | **+** | 0.0876 | 0.1246 | -0.0065 | 0.9081 |
| % > 60 | **-** | -0.1261 | 0.0004 | -0.1288 | 0.0025 |
| Crude birth rate | **+** | 0.1794 | 0.0068 | 0.0436 | 0.7242 |
| Pandemic Start | **+** | -0.0827 | 0.0205 | -0.0600 | 0.0513 |
| Pandemic Peak | **+** | 0.0052 | 0.8677 | 0.0497 | 0.0525 |
| 2008 H3N2 dominance | **-** | 0.0442 | 0.9425 | 0.2513 | 0.6079 |
| 2008 sH1N1 dominance | **+** | 0.0442 | 0.9425 | 0.2513 | 0.6079 |
| 2009 H3N2 co-circulation | **+** | -0.1677 | 0.8004 | -0.6449 | 0.2305 |
| 2009 flu B co-circulation | **+** | 0.1596 | 0.7866 | -0.0887 | 0.8525 |
| Lower respiratory deaths | **+** | -0.0014 | 0.9003 | 0.0058 | 0.5082 |
| All registered take offs | **+** | 0.0001 | 0.6944 | 0.0001 | 0.5173 |
